# Pilot RCT comparing low-dose naltrexone, gabapentin and placebo to reduce pain among people with HIV with alcohol problems

**DOI:** 10.1371/journal.pone.0297948

**Published:** 2024-02-26

**Authors:** Judith I. Tsui, Sarah L. Rossi, Debbie M. Cheng, Sally Bendiks, Marina Vetrova, Elena Blokhina, Michael Winter, Natalia Gnatienko, Miroslav Backonja, Kendall Bryant, Evgeny Krupitsky, Jeffrey H. Samet

**Affiliations:** 1 Department of Medicine, Division of General Internal Medicine University of Washington School of Medicine/Harborview Medical Center, Seattle, Washington, United States of America; 2 Department of Medicine, Section of General Internal Medicine, Boston Medical Center, Clinical Addiction Research and Education (CARE) Unit, Boston, Massachusetts, United States of America; 3 Department of Biostatistics, Boston University School of Public Health, Boston, Massachusetts, Unites States of America; 4 Pavlov University, St. Petersburg, Russian Federation; 5 Biostatistics and Epidemiology Data Analytics Center, Boston University School of Public Health, Boston, Massachusetts, United States of America; 6 National Center for Complementary and Integrative Health, National Institutes of Health, Bethesda, Maryland, United States of America; 7 HIV/AIDS Research, National Institute on Alcohol Abuse and Alcoholism, National Institutes of Health, Bethesda, Maryland, United States of America; 8 Department of Addictions, V.M. Bekhterev National Medical Research Center for Psychiatry and Neurology, St. Petersburg, Russian Federation; 9 Department of Medicine, Section of General Internal Medicine, Boston University School of Medicine/Boston Medical Center, Clinical Addiction Research and Education (CARE) Unit, Boston, Massachusetts, United States of America; 10 Department of Community Health Sciences, Boston University School of Public Health, Boston, Massachusetts, United States of America; Kamuzu University of Health Sciences, MALAWI

## Abstract

**Background:**

To estimate the effects on pain of two medications (low-dose naltrexone and gabapentin) compared to placebo among people with HIV (PWH) with heavy alcohol use and chronic pain.

**Methods:**

We conducted a pilot, randomized, double-blinded, 3-arm study of PWH with chronic pain and past-year heavy alcohol use in 2021. Participants were recruited in St. Petersburg, Russia, and randomized to receive daily low-dose naltrexone (4.5mg), gabapentin (up to 1800mg), or placebo. The two primary outcomes were change in self-reported pain severity and pain interference measured with the Brief Pain Inventory from baseline to 8 weeks.

**Results:**

Participants (N = 45, 15 in each arm) had the following baseline characteristics: 64% male; age 41 years (SD±7); mean 2 (SD±4) heavy drinking days in the past month and mean pain severity and interference were 3.2 (SD±1) and 3.0 (SD±2), respectively. Pain severity decreased for all three arms. Mean differences in change in pain severity for gabapentin vs. placebo, and naltrexone vs. placebo were -0.27 (95% confidence interval [CI] -1.76, 1.23; p = 0.73) and 0.88 (95% CI -0.7, 2.46; p = 0.55), respectively. Pain interference decreased for all three arms. Mean differences in change in pain interference for gabapentin vs. placebo, and naltrexone vs. placebo was 0.16 (95% CI -1.38, 1.71; p = 0.83) and 0.40 (95% CI -1.18, 1.99; p = 0.83), respectively.

**Conclusion:**

Neither gabapentin nor low-dose naltrexone appeared to improve pain more than placebo among PWH with chronic pain and past-year heavy alcohol use.

**Clinical trial registration:**

ClinicalTrials.gov (NCT4052139).

## Introduction

Chronic pain is estimated to impact between 20–40% of people globally [1, 2] and can worsen quality of life [3], impacting clinical care, employment [4, 5], involvement in society [6], and mental health [6–9]. Among people with HIV (PWH), chronic pain prevalence estimates range from 25 to 90%, suggesting that chronic pain may be more common among PWH compared to persons without HIV, although prevalence may vary broadly by study population and setting [10–13]. Chronic pain among PWH can lead to worse HIV outcomes due to reduced treatment adherence and retention in care [4, 12, 14].

Opioids are more likely to be prescribed for pain and at higher doses to PWH compared to persons without HIV, which may place them at greater risk for opioid use disorders [15]. Previous work has shown that PWH with pain are more likely to report using illicit drugs [16, 17] and drink heavily [18]. The Infectious Disease Society of America shared guidelines in 2017 recommending against prescribing opioid analgesics as first line treatment for PWH reporting chronic pain [19]. There is a need for non-opioid treatments for pain among PWH, particularly among those with alcohol use disorders (AUDs), which is a known risk factor for prescription opioid misuse [20].

Previous studies have suggested benefits from low-dose naltrexone (i.e., less than five mg) for chronic pain conditions (such as fibromyalgia and complex regional pain syndromes) that are postulated to be driven by neuro-inflammation [21, 22]. Furthermore, naltrexone at higher doses (50mg or more) is established as an effective treatment of AUD [23]. Associations between hazardous alcohol use and chronic pain have been well-described, including among PWH [18, 24], and can be explained by underlying neurobiological mechanisms that are reinforcing and bi-directional [25, 26]. Thus, opioid receptor antagonists are an intriguing class of medications for persons with chronic pain and heavy alcohol use. In a previous pilot study, we looked at the tolerability and safety of two opioid receptor antagonists among PWH with chronic pain and heavy drinking based on NIAAA criteria [27]. We found that low-dose naltrexone (LDN) was tolerable and safe, but partial kappa-antagonist nalmefene was not [27].

In the present study, we looked at gabapentin and LDN in reducing pain. Gabapentin is one of the most common off-label medications used to treat chronic pain in the U.S. [28]. It is licensed to treat post-herpetic neuralgia and seizures, but is more broadly used as an alternative for opioid medications despite lack of evidence of effectiveness for chronic pain in general, which is most often musculoskeletal in nature [29]. Like naltrexone, gabapentin also has demonstrated effectiveness for AUDs in some studies [30, 31].

We conducted a pilot three-arm, double-blinded, randomized controlled trial of LDN and gabapentin versus placebo to estimate the effects of each of two medicines, LDN and gabapentin, on pain, alcohol use, inflammatory biomarkers, and measures of HIV progression, among PWH with chronic pain and heavy alcohol use.

## Material and methods

### Objective and study design

This PETER PAIN study was a double-blinded, randomized, three-arm parallel design pilot study to estimate the effects of LDN and gabapentin, each compared to placebo among PWH with heavy alcohol use and chronic pain. The two primary outcomes were change in self-reported pain severity and pain interference measured with the Brief Pain Inventory (BPI) from baseline to eight-weeks (end of treatment). Secondary outcomes were changes in cold pain tolerance, heavy drinking days, measures of HIV control, and inflammatory biomarkers from baseline to eight-weeks. Determination of the 8-week follow-up period was based on a prior review of studies on LDN which recommended 2 months of treatment to obtain an estimate of efficacy [22]. The target enrollment was 45 participants, 15 in each arm, followed for 12 weeks. The two primary comparisons of interest were LDN vs. placebo and gabapentin vs. placebo.

### Participants and recruitment

We recruited 45 participants between January and September 2021 from previous research cohorts, as well as from local non-governmental organizations, major HIV hospitals, and addiction hospitals in St. Petersburg, Russia. Inclusion criteria included: 18 years or older; HIV-positive (confirmed from testing or previous study records); chronic pain (present ≥three months) of moderate to severe intensity (moderate, severe, or very severe response to “How much bodily pain have you had during the last week?” and answered yes to “Do you have bodily pain that has lasted for more than three months?”); heavy drinking in the past year (based on NIAAA criteria: >14 standard drinks per week/>4 drinks in a day for men; seven drinks in the past week/>three drinks in a day for women); if female, negative pregnancy test and willing to use birth control while taking study medication; contact information for two contacts; address within 100 kilometers of St. Petersburg; possession of a telephone; able and willing to comply with all study procedures. Exclusion criteria included: not fluent in Russian; cognitive impairment resulting in inability to provide informed consent based on research assessor (RA) assessment; known active tuberculosis or current febrile illness; breastfeeding; known uncontrolled psychiatric illness; current suicidal ideation; history of hypersensitivity to naltrexone, gabapentin, or naloxone; current use (past week) of illicit or prescribed opiates as documented by either self-report or positive urine drug test; unwilling to abstain from opiates during the treatment period; current use of neuroleptics; history of seizure disorder; known liver failure, aspartate or alanine aminotransferase (ALT/AST) levels greater than 5 times the upper limit of normal; creatinine clearance (CrCl) less than 60 milliliters per minute; history of Raynaud’s disease; planned surgeries in the next three months; enrolled in another HIV and/or substance use medication intervention study; taking naltrexone, gabapentin, or pregabalin in the past 30 days; and diagnosis of chronic obstructive pulmonary disease because of recent warning of increased risk for respiratory depression with gabapentin [32].

Screening took place in-person, with some participants screened first over the phone. Trained RAs obtained verbal consent for screening each of the potential participants. If eligible on initial screening and after written informed consent was signed, potential participants received a pregnancy test (if female), urine drug test, rapid HIV test if no documentation available, and blood draw for liver function testing. The Institutional Review Boards (IRB) of Boston University Medical Campus and First St. Petersburg Pavlov State Medical University approved the PETER PAIN study. A Data Safety and Monitoring Board (DSMB) reviewed adverse events and study progress twice per year. The study was registered with ClinicalTrials.gov (NCT4052139).

### Naloxone challenge

At baseline after completing informed consent/enrollment, participants received the naloxone challenge to assess the safety of prescribing LDN. The naloxone challenge included a urine drug test and, if negative, administration of 0.8mg of naloxone intramuscularly. RAs observed participants for signs of opioid withdrawal for up to 20 minutes. Participants were allowed to return in the future to repeat the test if they failed the naloxone challenge.

### Intervention and randomization

After successfully completing the naloxone challenge, participants underwent the study interview assessment, cold pressor testing, and phlebotomy. They were then randomized to either daily LDN (4.5mg), gabapentin (up to 1800mg), or placebo with a 1:1:1 allocation ratio using permuted blocks, to ensure equal numbers in each arm. Participants and all research staff were blinded to the randomization arm.

Gabapentin was titrated up to a maximum dose of 1800mg per day based on standard FDA recommended dosage for neuropathic pain. Participants were titrated from 300mg daily (week one), to 900mg daily (week two), to 1800mg daily (week three), and remained at 1800mg until week eight where they tapered down to 900mg daily on days five through seven of week eight. Given that gabapentin was only available in 300mg capsules, participants were instructed to take one capsule per day in week one, one capsule three times per day in week two, two capsules three times per day in weeks three through seven and days one through four of day eight, and one capsule three times per day in days five through seven of week eight. In order to keep the blind among research staff and participants, those in the LDN and placebo arms received additional placebo capsules to match the capsule schedule of the gabapentin arm. All capsules looked identical and were delivered in a prefilled weekly pill box.

### Study visits and measures

Participants came to Pavlov State Medical University for study visits at baseline, four, eight, and 12-weeks post-randomization and came in for shorter medication check-ins at weeks one, two, six, and seven. Compensation was provided for each visit. Pregnancy tests were administered baseline through week seven to ensure that participants did not become pregnant while on study medication. Blood was collected for ALT/AST testing at week four. Blood was also collected at baseline and week eight for inflammatory biomarkers, CD4 cell count, and HIV viral load (HVL) testing. Study assessments were administered by RAs.

Data collected during the assessments included the following: demographics, alcohol use via timeline followback [33], alcohol use via AUDIT [34], alcohol craving [35], depressive symptoms [36, 37], anxiety [38], ART use and adherence [39], comorbidities [40], HIV symptoms [41], prescription and non-prescription medication use, pain [42], adjunct treatments of pain, drug use [43, 44], and general health via the Veterans RAND 12 Item Health Survey (VR-12) [45, 46]. The following data were also collected at follow-up visits: medication adherence [39], medication satisfaction [47], and medication tolerability [48].

Cold pressor test as a measure of hyperalgesia was used to assess cold pain threshold and cold pain tolerance at baseline, four, eight, and 12-week visits. The cold pressor test is an established tool to measure nociceptive responses and provides an experimental measurement of pain sensitivity/hyperalgesia. RAs instructed participants to place their dominant arm in a cold water (0–2°C) bath until their hand was covered with the ice water, and to indicate when they first perceived pain, to rate their pain on a scale of zero (low) to 100 (worst pain), and to remove their arm when the pain became intolerable.

Blood specimens were tested for the following at baseline and eight-weeks: CD4 cell count, HVL, three pro-inflammatory biomarkers (IL-6, IL-1β, and TNF-α), and one anti-inflammatory biomarker (IL-10). The biomarker levels were measured using commercially available enzyme-linked immunosorbent assay kits (R&D Systems Inc.).

Participants were instructed to return any unused study medication to every post-baseline study visit. The returned study medication was used to assess medication adherence via capsule counts–RAs counted and recorded the number of remaining capsules. In addition, medication adherence was measured through self-report via a visual analog scale (VAS). Participants were asked via a VAS ruler the percentage of study medication taken in the past week for the one, two, six, seven, and eight-week visits and the past two weeks for the four-week visit. For satisfaction, we report the global satisfaction score from the treatment satisfaction questionnaire for medication [47].

### Adverse events

At each study visit, RAs reviewed a medication symptom checklist (S1 File) that included well-known side effects of gabapentin and LDN, beginning with the symptoms reported at previous study visits.

Any event that met the criteria for an adverse event (AE), serious adverse event (SAE), or unanticipated problem (UP) was recorded. Every six months, any AEs, SAEs, and UPs were reported to the DSMB.

### Primary outcomes

**Fig 1:**
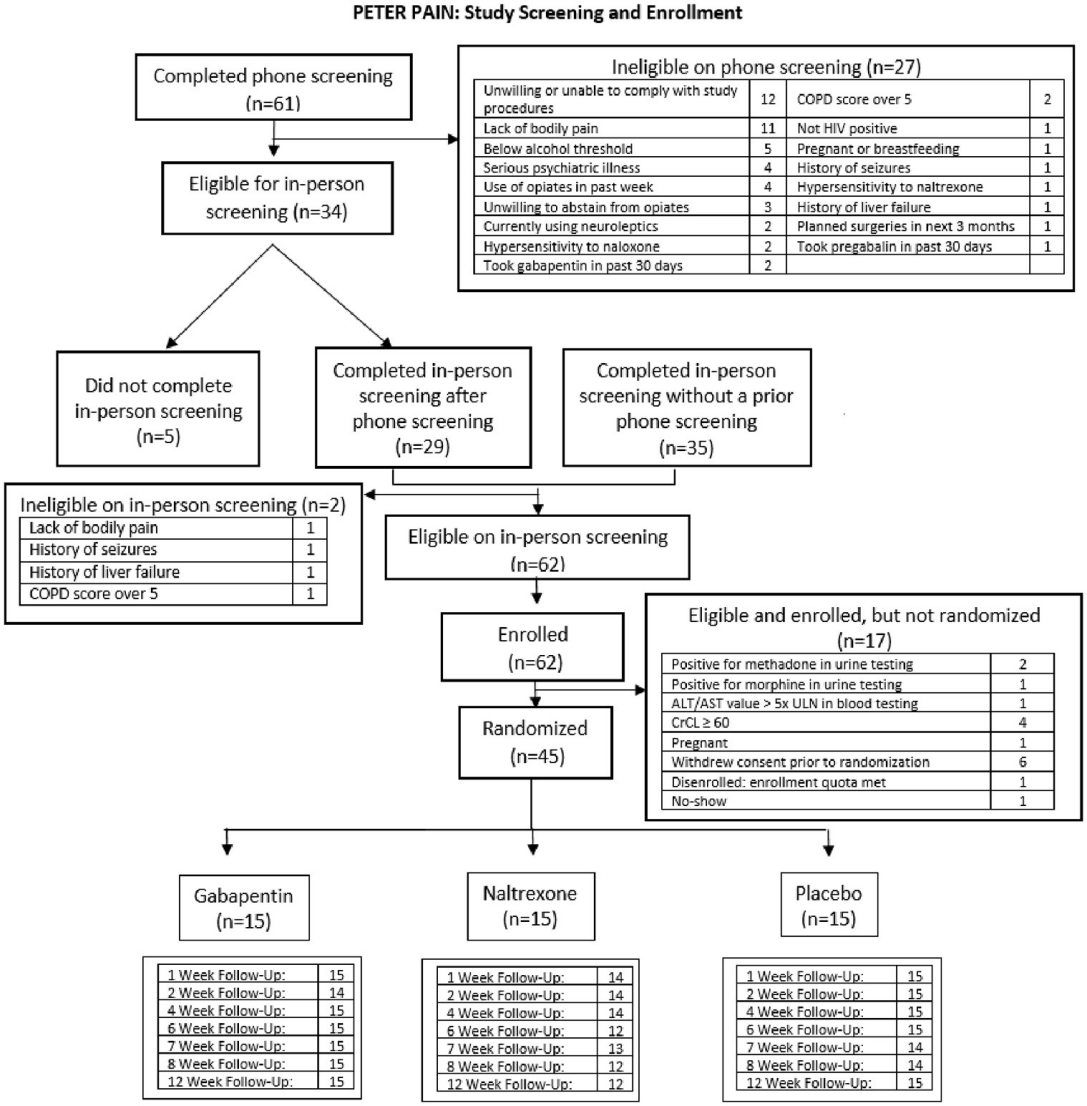
CONSORT flow diagram.

The two primary outcomes for this study were change in pain severity and pain interference between baseline and the eight-week visit, measured via the brief pain inventory (Fig 1) [42]. Pain severity was assessed as an average of four questions that asked about your worst pain, least pain, and average pain of the past seven days as well as your pain now, with scores ranging from “no pain (zero)” to “pain as bad as you can imagine (10).” Pain interference was assessed as an average of seven questions of potential interference of the past seven days (general activity, mood, walking ability, normal work, relations with other people, sleep, and enjoyment of life), with scores ranging from “does not interfere (zero)” to “interferes completely (10).” Change was calculated by subtracting the score at baseline from the score at week eight.

### Secondary outcomes

The secondary outcomes of this study were: 1) change in cold pain tolerance from baseline to week eight assessed using number of seconds participants could keep their arm in the cold water bath; 2) change in percentage of heavy drinking days in the past 30 days from baseline to week eight; 3) change in CD4 cell count from baseline to week eight. To explore inflammation as an underlying mechanism for changes in pain, we also examined changes in inflammatory biomarkers (IL-6, IL-10, IL-1β, and TNF-α) from baseline to week eight; these biomarkers were chosen on the basis of prior literature [49–54].

### Statistical analysis

Baseline characteristics of the randomization groups were described with proportions for dichotomous and categorical variables and means, standard deviations, medians, and interquartile ranges for continuous variables. Balance between randomization groups was assessed via chi-square or Fisher’s Exact test and ANOVA or Kruskal-Wallis tests as appropriate. Analyses for the PETER PAIN trial were conducted using the intention-to-treat approach; all randomized participants were included in the analysis. The primary analysis used linear regression models to estimate effects of LDN versus placebo and gabapentin versus placebo. We also performed pairwise comparisons between groups, adjusting for the multiple comparisons using the Hochberg sequential test procedure. We assessed model fit using residual plots. We accounted for missing data using multiple imputation by predictive mean matching. The variables used for imputation were randomization group, age, gender, percentage of heavy drinking days in the past month at baseline, lifetime opioid use, baseline values of all outcomes, and any available follow-up values for the outcome being imputed. Analyses of secondary outcomes were conducted using a similar approach as described for the primary outcomes. HVL, tolerability, medication discontinuation, adherence, and medication satisfaction were only examined descriptively. All analyses were conducted using SAS 9.4 (SAS Institute, Cary NC).

The purpose of the pilot trial was to generate preliminary data for a larger study to demonstrate efficacy. Thus, estimation rather than testing was the main objective of the research. Nevertheless, to define the limits of the study, power calculations for the planned sample size indicated that the minimum detectable difference in change in pain severity between baseline and 8 weeks that the study could detect with 80% power was 3.8 for either of the two primary comparisons of interest, and the 95% confidence interval for the mean difference in change in pain severity for either intervention arm vs. placebo was estimated to be no wider than 4.7, based on standard deviation estimates from prior literature [22]. Recruitment numbers also took into account perceived recruitment feasibility given the specific eligibility criteria.

## Results

Study participants had a mean age of 41 years (SD 7.0), the majority were male (64.4%), and about half had a lifetime history of opioid use (Table 1). Baseline mean pain severity was 3.2 (SD 1.3) and mean pain interference was 3.0 (SD 2.0), with scores ranging from zero to 10. One-fifth of participants reported depressive symptoms based on the CES-D (score of 16 or higher) [36, 37], and about one-quarter reported anxiety based on the GAD-7 (27.0% with a score of five or higher) [38]. Over half (60.0%) reported past year harmful and hazardous drinking via the AUDIT (score of eight or higher) [34]. The vast majority of participants (91.1%) had an undetectable HVL (<250 copies/mL) at baseline and mean (SD) CD4 cell count was 791 (331) cells/mm^3^. Almost all participants reported current ART use at baseline (93.3%). The average physical and mental component scores of the VR-12 were 40.8 (SD 7.1) and 47.1 (SD 9.6), respectively. Overall, groups appeared balanced across baseline characteristics (Table 1), with the exception that the gabapentin group appeared to have a higher proportion of persons who were married or in long-term partnerships.

**Table 1:** Baseline characteristics of PETER PAIN study participants: People with HIV, chronic pain, and heavy alcohol use (n = 45).

Characteristic	Overall (n = 45)	Gabapentin (n = 15)	Naltrexone (n = 15)	Placebo (n = 15)
Age, mean (SD), years	41 (7)	41 (7)	40 (6)	41 (7)
Male, n (%)	29 (64.4%)	10 (66.7%)	9 (60.0%)	10 (66.7%)
Education (9 grades or more), n (%)	45 (100.0%)	15 (100.0%)	15 (100.0%)	15 (100.0%)
Married/living with partner/in long-term relationship, n (%)	22 (48.9%)	11 (73.3%)	5 (33.3%)	6 (40.0%)
Harmful or hazardous drinking, n (%)^a^	27 (60.0%)	9 (60.0%)	10 (66.7%)	8 (53.3%)
Number of heavy drinking days in past 30 days, mean (SD)	2 (4)	2 (3)	2 (4)	2 (4)
Past month heavy drinking days, mean % (SD)	7.6 (12.8)	6.7 (9.4)	8.0 (14.6)	8.0 (14.5)
Lifetime opioid use, n (%)	25 (55.6%)	9 (60.0%)	9 (60.0%)	7 (46.7%)
Pain severity, mean (SD) Median (IQR)^b^	3.2 (1.3) 3.0 (2.3, 4.0)	3.1 (1.3) 2.8 (2.0, 4.5)	3.2 (1.4) 3.3 (2.8, 3.8)	3.3 (1.5) 2.8 (2.5, 4.0)
Pain interference, mean (SD) Median (IQR) ^b^	3 (2) 2 (1, 4)	3 (2) 3 (1, 4)	3 (2) 2 (1, 3)	3 (2) 1 (1, 4)
Cold pain threshold, mean (SD) Median (IQR)	17 (13) 12 (8, 19)	15 (13) 9 (7, 20)	21 (17) 13 (8, 32)	14 (6) 15 (11, 17)
Cold pain tolerance, mean (SD) Median (IQR)	37 (33) 25 (18, 45)	33 (25) 22 (14, 52)	42 (33) 37 (18, 54)	36 (41) 25 (17, 35)
IL-6 (pg/ml) mean (SD) Median (IQR)	3.7 (0.5) 3.6 (3.3, 3.9)	3.7 (0.4) 3.7 (3.6, 3.9)	3.8 (0.7) 3.6 (3.3, 4.2)	3.5 (0.4) 3.5 (3.3, 3.7)
IL-10 (pg/ml) mean (SD) Median (IQR)	3.9 (0.7) 3.7 (3.4, 4.2)	3.9 (0.7) 3.7 (3.5, 4.2)	3.8 (0.7) 3.7 (3.3, 4.6)	3.9 (0.8) 3.5 (3.4, 4.2)
TNFa(pg/ml) mean (SD) Median (IQR)	6.0 (1.0) 5.9 (5.3, 6.6)	5.7 (0.9) 5.6 (5.3, 6.6)	5.7 (0.7) 5.6 (5.2, 6.3)	6.4 (1.2) 6.3 (5.3, 6.9)
IL-Ib (pg/ml) mean (SD) Median (IQR)	5.6 (0.6) 5.6 (5.2, 5.9)	5.6 (0.7) 5.6 (5.0, 6.3)	5.7 (0.5) 5.6 (5.4, 6.1)	5.5 (0.6) 5.6 (5.0, 5.9)
Currently on ART, n (%)	42 (93.3%)	15 (100.0%)	12 (80.0%)	15 (100.0%)
Undetectable HIV viral load, n (%) (<250 copies/mL)	41 (91.1%)	14 (93.3%)	12 (80.0%)	15 (100.0%)
CD4 cell count, mean (SD), cells/mm^3^	791 (331)	808 (290)	648 (273)	917 (381)
Depressive symptoms, n (%)^c^	9 (20.0%)	3 (20.0%)	3 (20.0%)	3 (20.0%)
Anxiety, n (%)^d^	5 (11.1%)	2 (13.3%)	2 (13.3%)	1 (6.7%)
Physical component score, mean (SD), median (IQR)^e^	40.8 (7.1) 42.9 (36.7, 45.6)	41.3 (7.2) 42.6 (38.5, 46.5)	38.1 (7.0) 38.2 (33.3, 44.6)	42.9 (6.7) 44.9 (39.6, 46.2)
Mental component score, mean (SD), median (IQR)^e^	47.1 (9.6) 49.4 (42.7, 54.7)	47.5 (8.8) 49.1 (45.4, 54.7)	44.4 (10.8) 46.0 (37.7, 51.9)	49.5 (9.1) 52.1 (43.9, 57.4)

^a^ AUDIT score 8+

^b^ BPI score range from 0 to 10

^c^ CES-D score 16+

^d^ Moderate to severe anxiety based on GAD-7

^e^ Physical and mental component scores of the Veterans RAND 12 Item Health Survey (VR-12)

Of the 59 participants who completed a phone screening, 32 were eligible for in-person screening; 34 completed an in-person screening without a phone screening. Of the 61 participants who completed an in-person screening, 45 were enrolled and randomized, 15 participants in each arm (Fig 1). During the study, one participant withdrew consent. The study blind was broken for one participant in the LDN group with a serious adverse event that required medical attention.

**Fig 2:**
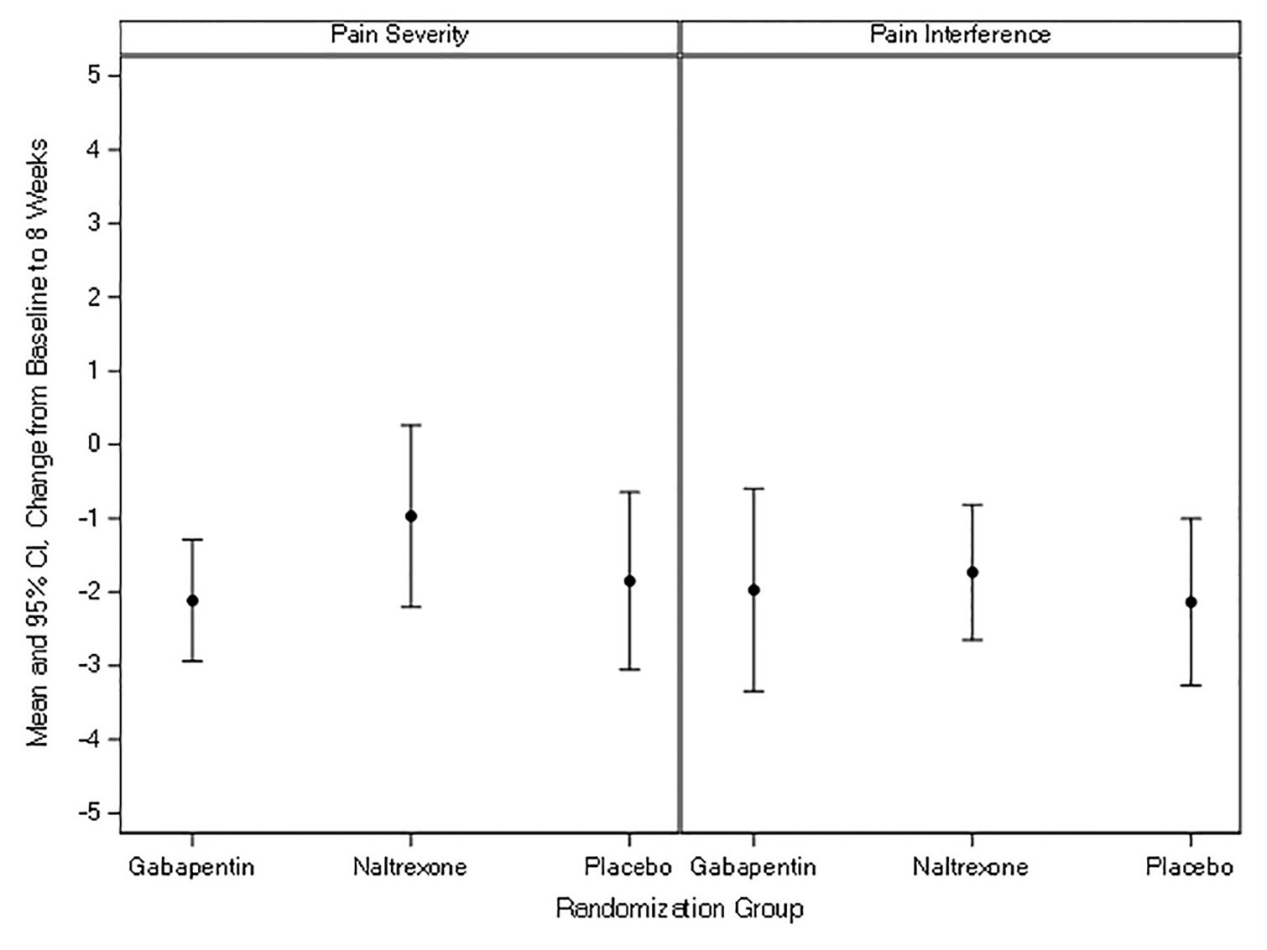
Mean change in pain severity and interference from baseline to 8 weeks.

**Fig 3:**
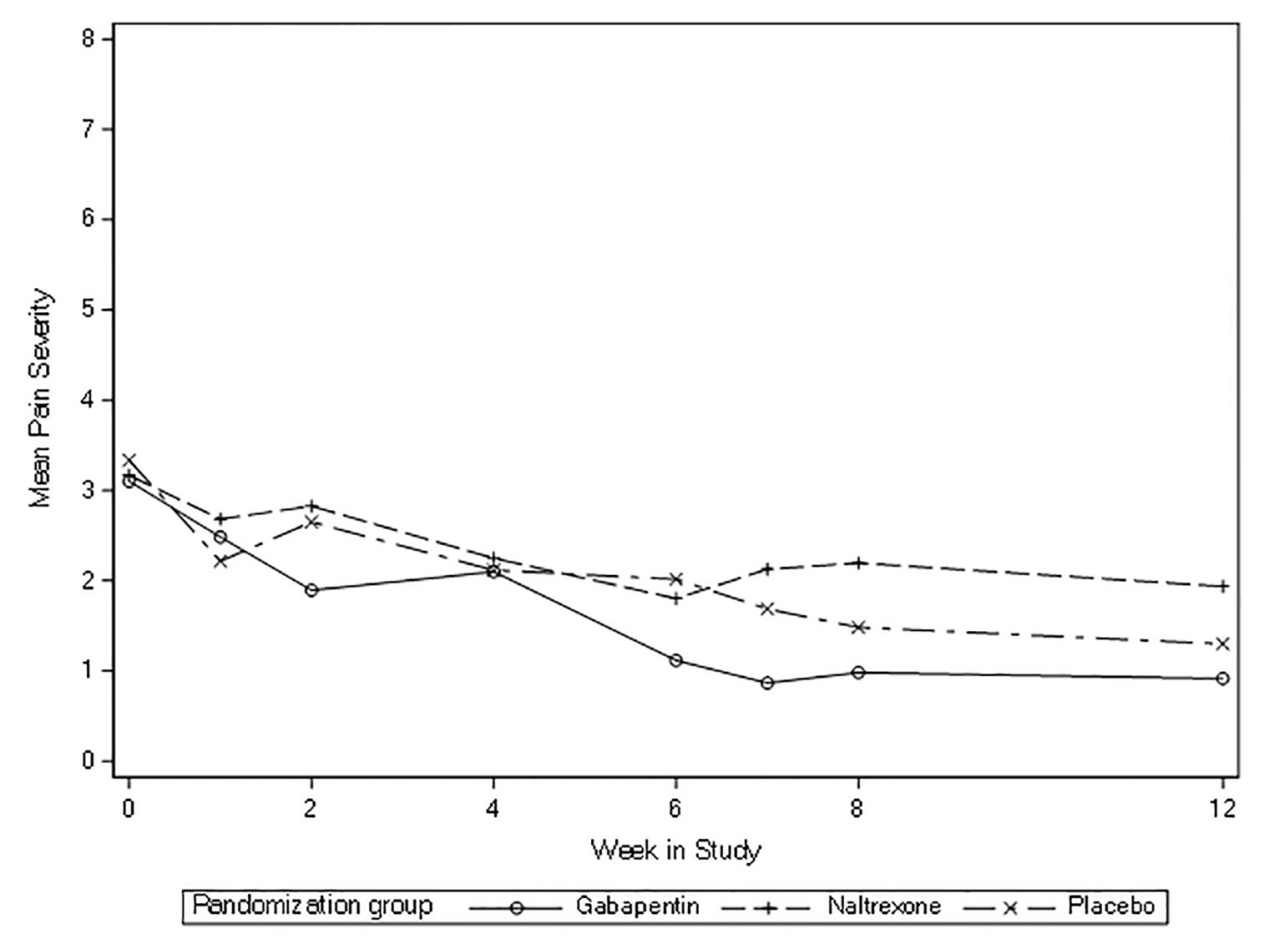
Mean pain severity overtime (baseline to 12 weeks).

**Fig 4:**
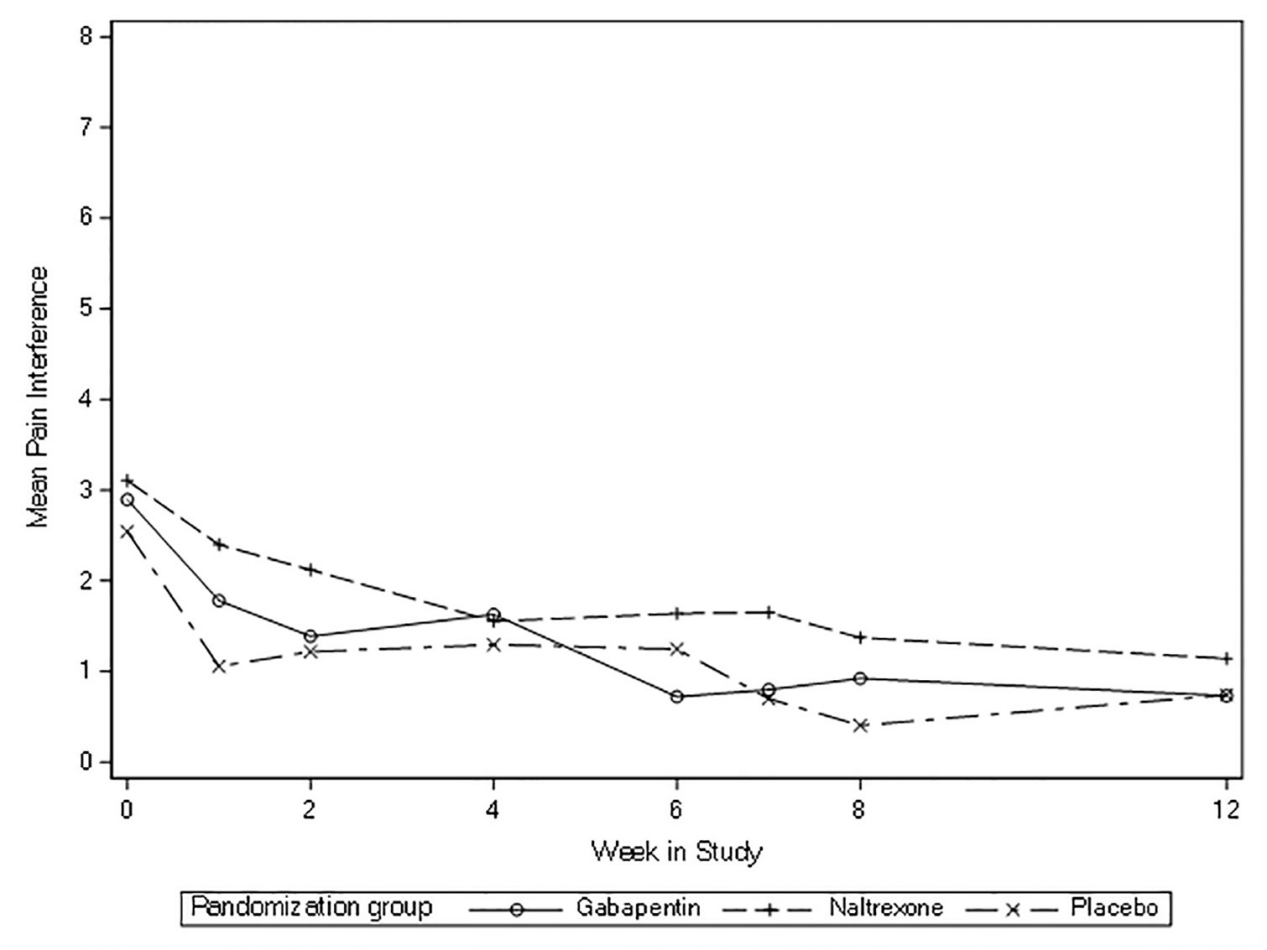
Mean pain interference overtime (baseline to 12 weeks).

All three groups had decreases in pain severity and interference between eight-weeks and baseline (Table 2, Fig 2). The mean change in pain severity from baseline to eight-weeks for gabapentin was -2.12, for LDN -0.97, and for placebo -1.85. For pain interference, the mean change from baseline to eight-weeks for gabapentin was -1.97, for LDN -1.73, and for placebo -2.14. There were no significant differences between groups: the mean difference for change in pain severity was -0.27 (95% confidence interval [CI] -1.76, 1.23; p = 0.73) for gabapentin vs. placebo and 0.88 (95% CI -0.7, 2.46, p = 0.55) for LDN vs. placebo. The mean difference for change in pain interference was 0.16 (95% CI -1.38, 1.71; p = 0.83) for gabapentin vs. placebo and 0.40 (95% CI -1.18, 1.99; p = 0.83) for LDN vs. placebo. None of these changes approached established cut-points for clinically important differences which are between two to three points [55, 56]. Across the 12 weeks of the study, pain severity and interference appeared to decrease with no substantial differences between groups (Figs 3 and 4).

**Table 2:** Main results: Changes in primary and secondary outcomes from baseline to 8 weeks in the PETER PAIN trial.

Outcome	Gabapentin	Naltrexone	Placebo	Gabapentin vs. Placebo Mean difference (95% CI)	p-value ^g^	Naltrexone vs. Placebo Mean difference (95% CI)	p-value ^g^
	**Mean Change from Baseline to Week 8**						
Pain severity	-2.12	-0.97	-1.85	-0.27 (-1.76, 1.23)	0.73	0.88 (-0.70, 2.46)	0.55
Pain interference	-1.97	-1.73	-2.14	0.16 (-1.38, 1.71)	0.83	0.40 (-1.18, 1.99)	0.83
Cold pain threshold	-1.13	-7.75	-1.40	0.27 (-8.94, 9.48)	0.95	-6.35 (-15.63, 2.92)	0.36
Cold pain tolerance	-3.33	-14.78	-3.15	-0.18 (-17.93, 17.57)	0.98	-11.62 (-29.93, 6.68)	0.43
IL-6 (pg/ml)	0.04	-0.12	0.29	-0.26 (-0.67, 0.15)	0.43	-0.42 (-0.86, 0.02)	0.19
IL-10 (pg/ml)	0.07	-0.13	-0.26	0.33 (-0.26, 0.91)	0.69	0.12 (-0.49, 0.74)	0.69
TNF-α (pg/ml)	0.47	0.27	0.21	0.26 (-0.59, 1.11)	0.89	0.06 (-0.83, 0.96)	0.89
IL-1β (pg/ml)	0.95	0.30	0.41	0.54 (-0.11, 1.19)	0.21	-0.10 (-0.77, 0.56)	0.76
% of past month heavy drinking days	-4.22	3.07	-4.63	0.41 (-11.09, 11.91)	0.94	7.70 (-4.02, 19.41)	0.44
CD4 count (cells/mm^3^)	-106.47	15.85	-52.13	-54.33 (-257.29, 148.63)	0.60	67.98 (-143.34, 279.30)	0.60

^g^ p-values are from t-tests

**Fig 5:**
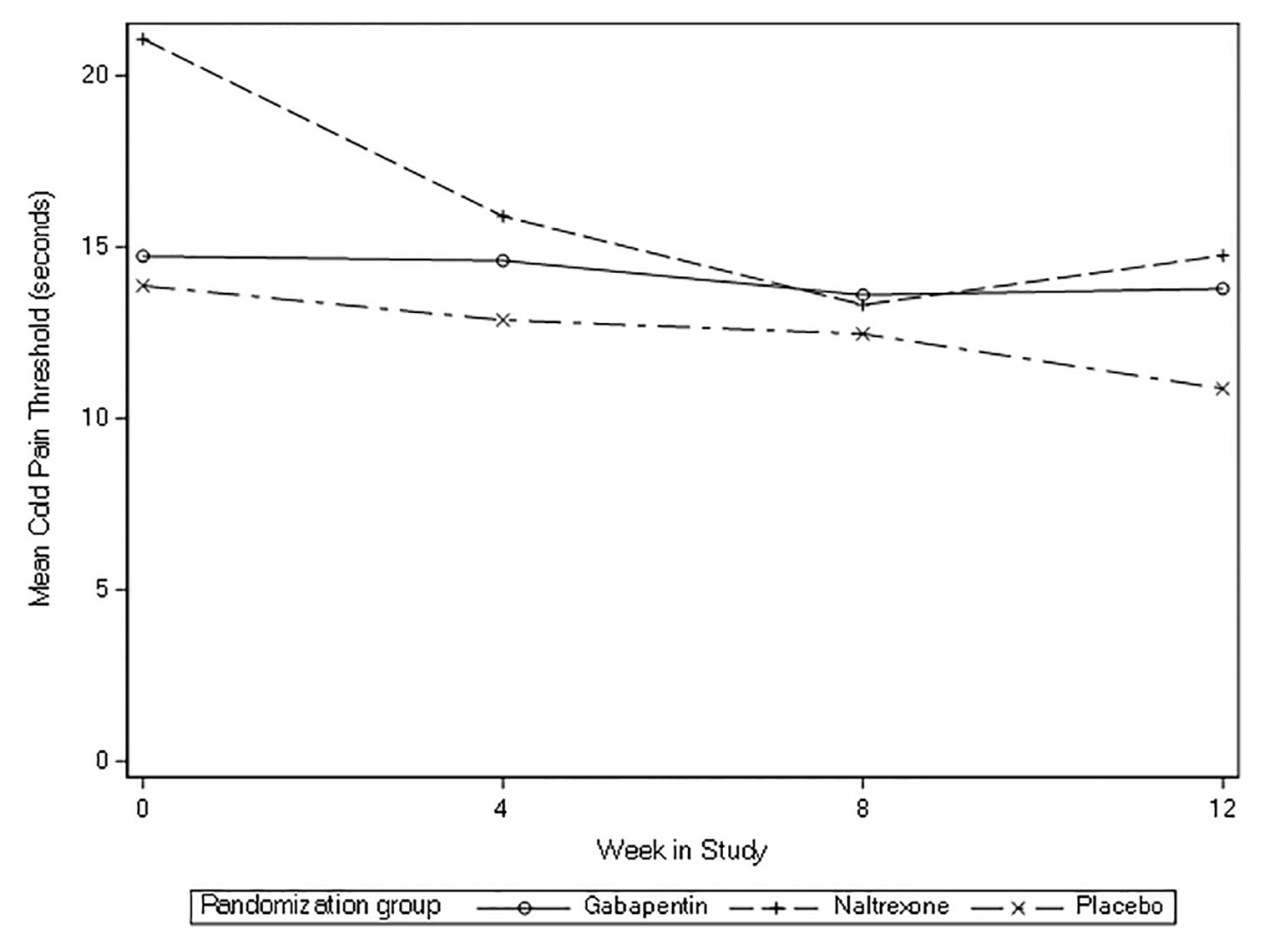
Mean cold pressor threshold overtime (baseline to 12 weeks).

**Fig 6:**
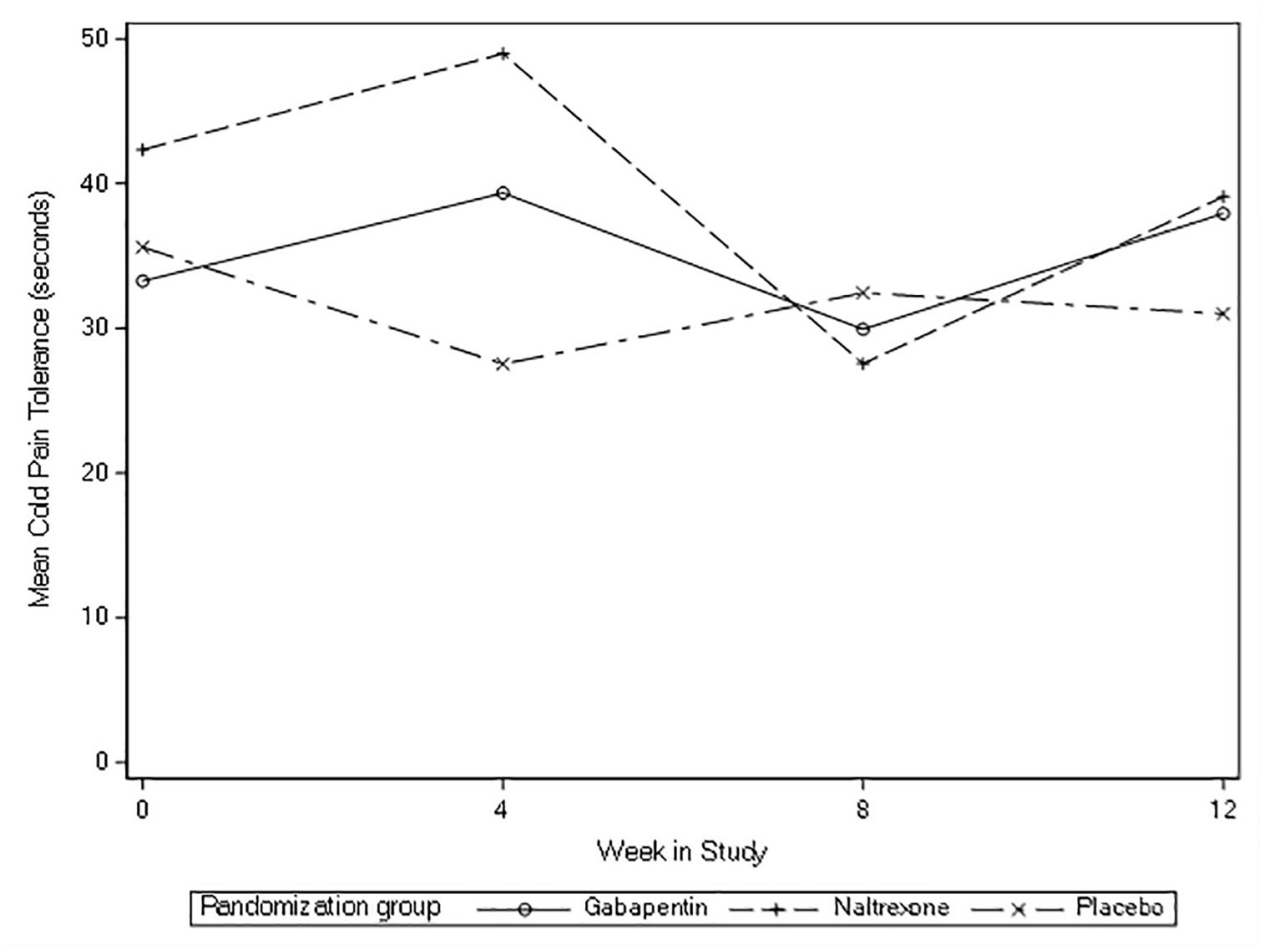
Mean in cold pressor tolerance overtime (baseline to 12 weeks).

For cold pressor secondary outcomes, all three groups had decreases in cold pain tolerance and threshold, but no substantial differences were detected for any comparisons of interest (gabapentin vs. placebo, LDN vs. placebo) at eight-weeks (Table 2). Across the 12 weeks of the study, cold pressor threshold and tolerance appeared to not change (Figs 5 and 6).

For all of the inflammatory biomarker outcomes (IL-6, IL-10, IL-1β, and TNF-α), we found no clinical or statistical differences for gabapentin vs. placebo or LDN vs. placebo (Table 2).

The gabapentin and placebo arms reported a slight decrease in mean change from baseline to eight-weeks in percentage of past month heavy drinking days (-4.22% and -4.63%, respectively), and the LDN arm reported a slight increase in mean change from baseline to eight-weeks in percentage of past month heavy drinking days (3.07%). The differences were not significantly different between arms (Table 2). There were also no clinical or statistical differences between groups in change in CD4 cell count between baseline and eight-weeks (Table 2).

In descriptive analyses of HVL suppression, most participants did not change their HVL suppression status between baseline and eight-weeks (97.4%).

Most participants found the medication tolerable using tolerability scale 0–100 ranging from 0 = cannot tolerate at all to 100 = tolerate perfectly well, reporting a mean (SD) of 91.0 (26.3) for gabapentin, 90.7 (25.0) for LDN, and 100.0 (0.0) for placebo over the course of the study (Table 3). There were seven permanent medication discontinuations, four in the gabapentin arm and three in the LDN arm. Participants reported relatively high adherence to the medication regimen throughout the course of the study, with overall adherence means (SD) of 74.4% (40.4) for gabapentin, 81.5% (32.9) for LDN, and 96.6% (4.9) for placebo, which stayed consistent throughout the study (Table 3). At eight-weeks, the mean (SD) satisfaction scores were 66.7% (17.6) for gabapentin, 52.6% (25.0) for LDN, and 58.7% (19.2) for placebo (Table 4). For adverse events, there were six events in the gabapentin arm, 13 in the LDN arm, and five in the placebo arm over the course of the study (Tables 5–7).

**Table 3:** Medication tolerability, discontinuations, adherence over the 8-week study period.

Variable		Gabapentin	Naltrexone	Placebo
Medication tolerability	Mean (SD)	91.0 (26.3)	90.8 (25.0)	100.0 (0.0)
	Median (IQR)	100.0 (100.0, 100.0)	100.0 (100.0, 100.0)	100.0 (100.0, 100.0)
Permanent treatment discontinuations	N (%)	4 (26.7%)	3 (20.0%)	0 (0.0%)
Adherence (VAS Score of 0–100)	Mean (SD)	74.4 (40.4)	81.5 (32.9)	96.6 (4.9)
	Median (IQR)	99.8 (18.0, 100.0)	99.1 (75.0, 100.0)	99.2 (93.3, 100.0)

**Table 4:** Medication satisfaction over 8-week study medication period.

Variable		Gabapentin	Naltrexone	Placebo
TSQM Global Satisfaction score, 2-week	N	14	13	15
	Mean (SD)	58.7 (15.9)	47.8 (21.5)	62.4 (19.6)
	Median (IQR)	57.1 (50.0, 71.4)	50.0 (50.0, 64.3)	57.1 (50.0, 78.6)
TSQM Global Satisfaction score, 4-week	N	13	12	15
	Mean (SD)	62.6 (17.8)	54.2 (21.9)	56.7 (13.1)
	Median (IQR)	57.1 (50.0, 78.6)	60.7 (50.0, 71.4)	57.1 (50.0, 71.4)
TSQM Global Satisfaction score, 6-week	N	13	11	15
	Mean (SD)	66.5 (15.8)	53.9 (18.2)	59.5 (17.4)
	Median (IQR)	71.4 (57.1, 78.6)	50.0 (50.0, 71.4)	57.1 (42.9, 71.4)
TSQM Global Satisfaction score, 8-week	N	12	11	14
	Mean (SD)	66.7 (17.6)	52.6 (25.0)	58.7 (19.2)
	Median (IQR)	71.4 (50.0, 78.6)	50.0 (50.0, 71.4)	57.1 (42.9, 71.4)

**Table 5:** Adverse events in gabapentin arm.

Body System	Event description	Mild	Moderate	Severe	Total
Gastrointestinal	Abdominal Pain	0	1	0	1
	Flatulence	0	1	0	1
Nervous system	Confusion	1	0	0	1
Infections and Infestations	Cold	0	1	0	1
Musculoskeletal and connective tissue	Burning in the legs	0	1	0	1
Psychiatric	Anxiety	0	0	1	1
Total		1	4	1	6

**Table 6:** Adverse events in low-dose naltrexone arm.

Body System	Event description	Mild	Moderate	Severe	Total
General	Fatigue	3	0	0	3
Gastrointestinal	Diarrhea	1	0	0	1
	Stomach Pain	1	0	0	1
	Loss of appetite	1	0	0	1
Nervous system	Dizziness	1	0	0	1
	Seizure	0	0	1	1
	Speech Impediment	0	1	0	1
	Tremor	1	0	0	1
Psychiatric	Sleepiness	0	1	0	1
	Insomnia	1	0	0	1
Cardiac	Hypertension	0	0	1	1
Total		9	2	2	13

**Table 7:** Adverse events in placebo arm.

Body System	Event description	Mild	Moderate	Severe	Total
General	Fatigue	1	0	0	1
Gastrointestinal	Loss of appetite	0	1	0	1
Skin	Redness of the skin of the upper body	1	0	0	1
Psychiatric	Insomnia	0	1	0	1
Reproductive System and Breast	Acute purulent non-lactation mastitis on the left side	0	0	1	1
Total		2	2	1	5

## Discussion

Among PWH with recent heavy alcohol use and chronic pain in the PETER PAIN randomized clinical trial, we did not detect clinical or statistical differences between LDN and placebo and gabapentin and placebo for any of the primary and secondary outcomes. All randomized groups experienced decreases in pain severity and interference, as well as decreases in cold pain threshold and tolerance at eight-weeks (primary study endpoint).

We found no notable clinical differences in the changes of inflammatory biomarkers between baseline and eight-weeks for any of the arms. Previous studies have shown reductions in pro-inflammatory IL-6, IL-1beta, and TNF-alpha and anti-inflammatory IL-10 from LDN [49, 50] and reductions in IL-6, IL-1β, and TNF-α and increases in IL-10 from gabapentin [51–54]. The present study’s results did not consistently align with previous studies, potentially due to low baseline values of these biomarkers.

These results are important to consider in the landscape of treating chronic pain, given that gabapentin has become the most common off-label prescription for pain in the U.S. [28]. While some studies have suggested benefits in treating pain from LDN [21, 22] and gabapentin [57, 58], others have not detected benefits and have suggested to carefully consider prescribing gabapentin [59–61]. Naltrexone has been approved for treatment for alcohol dependence since 1994 [23], while gabapentin has shown mixed results in treating alcohol dependence [30, 31, 62–64]. While LDN has shown to have minimal side effects in the literature [65], there have been reports of misuse of and safety issues with gabapentin [62, 66–68]. It is also notable that compared to placebo, participants in the LDN arm demonstrated worse cold pain tolerance thresholds (albeit non-significant) which is somewhat surprising given prior literature suggesting it can prevent opioid-induced hyperalgesia [69, 70]. However, these results should be interpreted cautiously given the small sample size and the impact of multiple comparisons in the overall study analyses. These results provide some preliminary evidence of LDN and gabapentin tolerability and adherence in the context of a clinical trial; data on patterns of long-term use of these medications in real-world settings should be a topic for future investigation.

The relatively low baseline scores of pain severity and pain interference in the PETER PAIN study warrant discussion about how pain is perceived and communicated. The International Association for the Study of Pain defines pain as “an unpleasant sensory and emotional experience associated with, or resembling that associated with, actual or potential tissue damage” [71]. Researchers have also commented on the need to consider social components of the experience of pain [72], including the influence of culture on pain communication, beliefs, and coping [73–76], as well as the impact of being part of a marginalized group [77–79]. A study from 2014 reported that people in Russia and Poland reported less frequent pain and analgesic use than people in Belgium, France, Germany, Great Britain, Italy, and Spain [80]. It is possible that the relatively low baseline scores for pain severity and interference were influenced by differing cultural perceptions of pain in Russia. Yet, the brief pain inventory was validated in Russia and was shown to be reliable and valid [42, 81]. It is also important to contextualize results within the local treatment landscape. There are 38 different medications used to treat pain as adjunctive therapy in Russia, including opioids, steroids, anti-depressants, and myorelaxants. These medications are only endorsed for treatment in the setting of high intensity pain. A Russian pain association endorsed gabapentin and pregabalin for pain management in patients with diabetic neuropathy [82].

There are a number of limitations with this study. First, this study was a pilot with a small sample size that was not designed to detect statistically significant differences. The goal of this pilot study was to estimate effects of gabapentin and LDN compared to placebo to assess whether a larger study of these medications for this indication was justified. We did not observe results that suggested any clinically meaningful differences exist between the active medication arms (gabapentin and LDN) and placebo. In fact, the LDN arm reported more pain over time compared to placebo. The gabapentin arm was titrated up to a maximum of 1800mg, a threshold that was based on consideration of safety (particularly given FDA issued a new “black-box” warning for respiratory depression and increase risk of overdose death with gabapentinoids alone and with opioids in 2019) [32] and feasibility of study procedures. It is possible that this was not a high enough dosage to see ideal effects, as in clinical practice it is not uncommon for patients to be prescribed doses to 3600mg daily. However, another RCT of gabapentin for chronic lower back pain that did titrate to 3600mg did not observe any differences in pain over time compared to placebo [83]. Of note, the gabapentin arm titrated down from 1800mg to 900mg in days five through seven of the final medication week (week eight), which may have impacted the results of the eight-week assessment. Another limitation of the study was the fact that the average baseline pain severity in the sample was relatively low which could lead to “floor” effects. However, we were able to see declines in pain over time in the sample overall, but there was no suggestion of clinically meaningful differences in changes over time between the active medications and placebo. Future studies should consider cut-points for eligibility criteria of the primary outcome (for example, a median baseline pain severity >3) to avoid such a limitation. This study took place in St. Petersburg, Russia, among only white men and women, potentially making the results not as generalizable for a different context. Participant demographics were relatively balanced across study arms except for marital/partnered status which was more common among participants in the gabapentin arm. As a consequence of the relatively small sample size of this pilot study, randomization did not appear to balance this participant characteristic. Some study strengths include high medication adherence and follow-up rates, as well as the RCT design with placebo arm.

## Conclusions

Chronic pain is a common consequence of HIV and can severely impact one’s quality of life [13]. Thus, it is vital to seek interventions that will decrease pain, and that are unlikely to result in misuse, especially among those who are at risk (e.g., met criteria for heavy drinking). Results are relevant for clinicians considering prescribing gabapentin or LDN to patients with HIV presenting with pain and/or heavy alcohol use. Although the gabapentin did not appear to improve pain more than placebo, it is important to acknowledge the proven analgesic effects of placebos [84]. Furthermore, these results should not detract from evidence that points to the effectiveness of naltrexone and gabapentin for treatment of AUD. Future endeavors may examine gabapentin at higher doses and restrict to patients with greater pain severity or certain phenotypes, as well as integration of behavioral interventions to improve the lives of PWH and heavy drinking living with chronic pain.

## Supporting information

S1 File Follow-up symptoms checklist. (DOCX)

S2 File CONSORT checklist. (DOC)

S3 File Protocol. (DOCX)
