# Supplementary material for: Pilot RCT comparing low-dose naltrexone, gabapentin and placebo to reduce pain among people with HIV with alcohol problems
Source: PLoS One. 2024 Feb 26;19(2):e0297948. doi: 10.1371/journal.pone.0297948 (PMC10896547; doi:10.1371/journal.pone.0297948)
Supplement: S1 File — (DOCX) [file pone.0297948.s001.docx]

# Supplementary Materials. Follow-Up Symptoms Checklist

Part 1. Symptom Checklist

I am going to ask you about some specific symptoms. For each one I would like for you to tell me whether you have experienced this since your last study visit.

| **Symptom** | **No Symptom** | **Mild** | **Moderate** | **Severe** | **Life-threatening** |
| --- | --- | --- | --- | --- | --- |
| Agitation and/or Irritability |  |  |  |  |  |
| Anger |  |  |  |  |  |
| Depressed mood |  |  |  |  |  |
| Anxiety (includes nervousness and panic attacks) |  |  |  |  |  |
| Insomnia and/or other sleep problems |  |  |  |  |  |
| Abnormal dreams and/or nightmares |  |  |  |  |  |
| Headaches |  |  |  |  |  |
| Dizziness/lack of coordination |  |  |  |  |  |
| Nausea and/or vomiting |  |  |  |  |  |
| Joint/muscle pain |  |  |  |  |  |
| Abdominal pain/cramps |  |  |  |  |  |
| Fatigue |  |  |  |  |  |
| Drowsiness |  |  |  |  |  |
| Difficulty speaking |  |  |  |  |  |
| Tremor |  |  |  |  |  |
| Jerky movements |  |  |  |  |  |
| Fever |  |  |  |  |  |
| Double vision |  |  |  |  |  |
| Swelling (legs or feet) |  |  |  |  |  |
| Unusual eye movement |  |  |  |  |  |
| Have you had any additional symptoms or health problems you would like to report?  1.  2.  3.  4. |  |  |  |  |  |

Part 2. Pregnancy (ASK IF FEMALE ONLY)

| **1. Are you currently pregnant?** | **0  No** | **1  Yes** | **3  I don’t know** |
| --- | --- | --- | --- |

| **IF IN PERSON: CONFIRMATORY URINE PREGNANCY TEST** | | |  |
| --- | --- | --- | --- |
| **2. Did participant test positive for pregnancy?** | **0  No** | **1  Yes** | **3  Phone** |

Part 3. Medical Visits

| **1. Have you seen a doctor since your last visit?** | **0  No** | **1  Yes** |
| --- | --- | --- |
| **Reason:** |  |  |
| **2. Have you visited an emergency room since your last visit?**  **IF YES, ASSESSOR TO USE RESPONSE TO PROBE AND RECORD MISSED DOSES ON ADHERENCE FORM AND COMPLETE SAE FORM** | **0  No** | **1  Yes** |
| **Reason:** |  |  |
| **3. Have you been hospitalized since your last study visit?**  **IF YES, ASSESSOR TO USE RESPONSE TO PROBE AND RECORD MISSED DOSES ON ADHERENCE FORM AND COMPLETE SAE FORM** | **0  No** | **1  Yes** |
| **Reason:** |  |  |
