# Supplementary material for: Pilot RCT comparing low-dose naltrexone, gabapentin and placebo to reduce pain among people with HIV with alcohol problems
Source: PLoS One. 2024 Feb 26;19(2):e0297948. doi: 10.1371/journal.pone.0297948 (PMC10896547; doi:10.1371/journal.pone.0297948)
Supplement: S3 File — (DOCX) [file pone.0297948.s003.docx]

STUDY PROTOCOL

Pilot Study of an Opioid-receptor Antagonist and Gabapentin to Reduce Pain and Inflammation among HIV-Infected Persons with Alcohol Problems – UH3 Component

Principal Investigators:

Jeffrey H. Samet, MD, MA, MPH – Boston Medical Center (contact)

Judith I. Tsui, MD, MPH – University of Washington Department of Medicine

NIAAA Award Number: UH3AA026193

Clinicaltrials.gov Registration: NCT04052139

Boston University Medical Campus IRB Protocol Number: H-39162

Protocol Version: February 26^th^, 2020

**TABLE OF CONTENTS**

**1. Introduction #**

**1.1 summary**…………………………………………………………………..………………….5

**2. Overview of study design**

**2.1 study aims** 5

**2.2 study outcomes**………………………………………………………………………..........6

**2.3 study design** …………………………………………………………………………………6

**2.4 study site**…………………………………………………………………..……………..…..7

**2.5 inclusion criteria**……………………………………………………..…………………....8

**2.6 exclusion criteria** **at study entry**…………………………………………..………….8

**2.7 recruitment goals**……………………………………………….………………...………9

2.7.a. sample size calculation and power…………………………………………..9

**3. INTERVENTION**

**3.1 intervention overview**……………………………………………………………………10

**3.2 randomization**………...……………………………………….……………………………11

**3.3 intervention**………………………………………………….…………………………..….11

3.3.a. naltrexone……………………………. …………………………………………12

3.3.b. Gabapentin..…………………………. …………………………………………...13

3.3.c PLACEBO…………………………………………………………………………….14

**3.4 medication considerations** ………………………………………..…………………...…15

3.4.a. symptom monitoring……………………………. ……………………………….15

3.4.b. adherence…………..……………………………. ……………………………….16

3.4.c. medication disbursement………………………. ………………………………16

3.4.d. lost or stolen study medication…..…………. ……………………….……...18

3.4.e. discontinuation of study medication……..…. ………………………………18

**3.5 schedule of data collection.**………………………………………..….……………….19

3.5.a. visit windows……….……………………………. ………………………………19

**3.6 data sources**……………………………………………………………..….……………..…21

3.6.a. questionnaires……….………………………….…. ……………………………21

3.6.b. urine……….……………………………. ……………………………………….. 21

3.6.c. blood..…….……………………………. …………………………………………21

3.6.d. cold-pressor testing..…….…………..…………………………………………22

3.6.e. daily pain diary..…….…………..……………….……………………………….22

**4. study procedures**

**4.1 recruitment**………………………………………….…………………..….……………….22

**4.2 screening**……………….…………………………….…………………..….……………..…22

**4.3 informed consent**……………….………………….…………………..….……….……..…23

**4.4 visit flow**……………….…………………………….…………………..….……………..…24

**4.5 quality assurance**……………….…………………………….…………..……………..….25

**4.6 compensation**……………….…………………………….…………………..….……………25

**4.7 retention**……………….…………………………….…………………..….…………………25

**5. assessments**

**5.1 baseline assessment**………………………………………….…………….……………...…26

**5.2 follow-up assessments**……………………………………….…………….……….….....…27

5.2.a. medication visits assessments..……. …………………..………………….…….27

**6. participant safety**

**6.1 specification of safety parameters**……………………...…………….………….…..…28

**6.2 the methods and time for assessing, recording, and analyzing safety**

**parameters**..…………………………………………………………………………………..…..29

**6.3 procedures for eliciting reports of and for recording and reporting adverse**

**event and intercurrent illnesses**…………………………...…………….……………..….30

6.3.a. other events…………………...……. …………………..……………………….31

6.3.b. adverse event reporting….....……. …………………..………………………..32

6.3.c. sae reporting….....……. ………………………………..………………………...33

**6.4 the type and duration of the follow-up of subjects after adverse events**…....36

**6.5 unblinding protocol**………………………………………………………………………..36

**6.6 data safety and monitoring board**………………………………………………………37

**7. data management**

**7.1 data collection**……………….…………………………………………………………….38

**7.2 quality control process**…………………………………………………………………..38

**7.3 data security and confidentiality**………………………………………………………38

**7.4 web systems**……………………………………………………………………………………39

**8. statistical analysis**

**8.1 primary analyses**………………………..……………………………………………………40

**9. Repository**

**9.1 Purpose**…………...……………………..……………………………………………………40

**9.2 Stewardship**……...……………………..……………………………………………………40

**9.3 Release of Samples/Data**……...……………………..……………………………………41

**10. staff training** 42

**10. study contacts** 42

# **1. Introduction**

**1.1 summary**

Pain is a common co-morbidity for HIV-positive patients. Prevalence studies suggest that, on average, half of all HIV-positive persons suffer pain. Chronic pain can lead to heavy alcohol use among HIV-positive persons, which may in turn be a barrier to treatment/control of HIV and contribute to spread of HIV. Thus there is an urgent need to address pain among persons with HIV. We aim to perform a pilot RCT comparing low-dose naltrexone and gabapentin to placebo for improving pain, inflammation, and measures of HIV status among HIV-positive persons with chronic pain and heavy alcohol use. It is timely and relevant to conduct research on gabapentin. In this new era of judicious opioid prescribing it has emerged as one of the most commonly prescribed non-narcotic medications for pain. According to a recent review, gabapentin prescriptions increased from 44 to 68 million between 2013 and 2017, becoming the 10th most commonly prescribed medication in the United States.^1^ This is despite the fact that gabapentin is only FDA approved for “post-herpetic neuralgia” and the literature to support its use in other pain conditions, and generalized chronic pain, are limited. And yet, gabapentin has demonstrated benefits for treatment of alcohol use disorder,^2^ and therefore, like naltrexone, it could have a specific role for treating patients with chronic pain and unhealthy alcohol use.^3^

The specific aim of the research is to perform a 3-arm pilot randomized, double-blinded, placebo-controlled study of low-dose naltrexone and gabapentin vs. placebo among HIV-positive persons with heavy alcohol use and chronic pain to provide estimates of their effects on: 1) pain (both self-reported and experimental/cold pressor test; 2) inflammation (i.e., levels of inflammatory cytokines IL-6 and TNF-α); and 3) measures of HIV control (CD4 cell count and HIV viral load). We choose to conduct this research in St. Petersburg, Russia, given that: 1) patients are seldom on chronic opioids (which are contraindicated to use with opioid receptor antagonists) due to the unavailability of opioid agonist therapy for addiction and restricted use of opioids for pain; and 2) a high prevalence of heavy drinking and HIV exists in Russia.

During the UH2 component we found that K-opioid antagonist, nalmefene was not tolerable, leading us to modify the original research plan by replacing the nalmefene arm of the study with GABA analog, gabapentin. Given the US epidemic of opioid use disorders, new pharmacotherapies without addictive potential are desperately needed for HIV-positive persons with chronic pain and alcohol problems.

# **2. Overview of Study Design**

**2.1 study aims**

Our Specific Aim will compare effects of low-dose naltrexone and gabapentin to placebo among HIV-positive persons with heavy alcohol use and chronic pain:

**UH3 Aim**: To perform a 3-arm pilot, randomized, double-blinded, placebo-controlled study of low-dose naltrexone and gabapentin vs. placebo among HIV-positive persons with heavy alcohol use and chronic pain to provide estimates of their effects on 1) pain (both self-reported and experimental/cold pressor test); 2) inflammation (i.e., levels of inflammatory cytokines IL-6, IL-1β, IL-10, and TNF-α); and 3) measures of HIV control (CD4 cell count and HIV viral load).

**2.2 study outcomes**

**Primary Outcomes**: The primary outcome for this study is change in past week pain severity and pain interference (score 0-10) from baseline to week 8. Pain severity and interference will be measured using the Brief Pain Inventory (BPI).

**Secondary Outcomes**:

Changes in the following secondary outcomes between baseline and week 8 will also be compared among the 3 arms:

1) Biomarkers IL-6, IL-1β, 1L-10, and TNF-α. Levels of these pro-inflammatory markers will be measured on blood samples collected using commercially available enzyme-linked immunosorbent assay kits (R&D Systems).

2) Cold pain tolerance. This will be measured using the cold-pressor test; briefly it is the number of seconds a participant can keep hand submerged in cold water bath.

3) Percentage of past month heavy drinking days. We will use the Timeline Follow Back to assess drinks per day in the past 30 days. Using a calendar, people provide retrospective estimates of their daily drinking over the past 30 days from the interview date. Definitions of heavy drinking are based on NIAAA guidelines for at-risk drinking (i.e., drinking greater than 14 standard drinks per week/greater than 4 drinks in a day for men; greater than 7 drinks in the past week/greater than 3 drinks in a day for women).

4) Measures of HIV control: CD4 cell count and HIV viral load will be measured on participants.

**2.3 study design**

This is a randomized, double-blinded, 3-arm study of 45 participants with HIV, chronic pain (defined as any bodily pain of moderate to severe intensity lasting for ≥3 months), and past-year heavy alcohol use, defined by NIAAA criteria. The 45 participants will be randomly assigned to either naltrexone, gabapentin, or placebo, using permuted blocks, to ensure equal numbers in each arm. Patients and research staff will be blinded to randomization. Participants will be treated for 8 weeks, and then assessed for an additional 4 weeks post-treatment.

The study design and schedule for study visits and assessments is depicted in Figure 1. After consent and enrollment, participants will be randomly assigned to receive either low-dose naltrexone, gabapentin, or placebo. Participants will be randomized using permuted blocks, to ensure similar numbers within each arm. Patients and research staff will be blinded to randomization. Participants will be given 2 weeks of medication at baseline, 2-weeks, 4-weeks, and 6-week visits, with instructions to bring pill packs/leftover medications to the following visit. The schedule of study visits is as follows: baseline (pre-treatment), week 4 (on treatment), week 8 (on treatment), week 12 (post-treatment). At the 8 week visit, all assessments will occur (see Section 5). At week 4, we will assess for medication tolerability, discontinuation, treatment side effects, self-reported adherence to intervention medications, and pain (BPI) only. At week 12 (post-treatment), there will be an assessment of pain (BPI) and cold-pressor test to assess whether pain reverts back to pre-treatment levels. Weeks 1, 2, 6, and 7 will be short visits at which time a brief assessment of tolerability, side effects, and adherence will occur, and we will ensure that participants have their medications (e.g. have not been lost or stolen).

**Figure 1: Study Design (UH3 Phase)**

**Screening, Enrollment, & Baseline Assessment**

***Randomization N=45***

**Arm 1 N=15:**

4.5mg Naltrexone

**Arm 2 N=15:**

1800mg Gabapentin (Titrated from 300mg to reach 1800mg in Week 3)

**Arm 3 N=15**

Placebo

**Study visits at 4, 8, and 12 weeks**

**Short medication visits at 1, 2, 6, and 7 weeks**

**2.4 study site**

Recruitment, enrollment, and all study visits will take place at the Laboratory of Clinical Pharmacology of Addictions at the First St. Petersburg Pavlov State Medical University (PSMU) in St. Petersburg, Russia. PSMU is the major educational, scientific, and clinical medical institution for northwestern Russia. If unable to recruit an adequate sample size at these sites, we will broaden our recruitment methods to include other clinical sites of care for HIV-positive persons throughout St. Petersburg. Recruitment beyond Pavlov State Medical University (PSMU) will occur at Russia ARCH network hospitals including the major clinical HIV hospitals (Botkin Infectious Disease Hospital, Leningrad Regional AIDS Center, St. Petersburg City AIDS Center) and addiction hospitals (Leningrad Regional Center for Addictions, St. Petersburg City Addiction Hospital). Blood specimens will be processed and analyzed at ImmunoBioService (IBS) under the direction of Dr. Sergei Selkov.

**2.5 inclusion criteria**

To be eligible to participate in the trial, participants will need to meet the following inclusion criteria:

1. 18 years or older
2. HIV-positive
3. Chronic pain (present ≥3 months) of moderate to severe intensity
4. Heavy drinking past year (Based on NIAAA criteria: > 14 standard drinks per week/ > 4 drinks in a day for men; > 7 drinks in the past week/ > 3 drinks in a day for women.)
5. If female, negative pregnancy test and willing to use adequate birth control
6. Provision of contact information for 2 contacts to assist with follow-up
7. Address within 100 kilometers of St. Petersburg
8. Possession of a telephone (home or cell)
9. Able and willing to comply with all study protocols and procedures

**2.6 exclusion criteria at study entry**

1. Not fluent in Russian
2. Cognitive impairment resulting in inability to provide informed consent based on research assessor (RA) assessment
3. Known active TB or current febrile illness
4. Breastfeeding
5. Known uncontrolled psychiatric illness (such as active psychosis)
6. Current suicidal ideation
7. History of hypersensitivity to naltrexone, gabapentin, or naloxone
8. Current use (past week) of illicit or prescribed opiates as documented by either self-report or positive urine drug test
9. Unwilling to abstain from opiates during the treatment period
10. Current use of neuroleptics
11. History of seizure disorder
12. Known liver failure
13. ALT/AST levels >5x normal
14. CrCl < 60 mL/min
15. History of Raynaud’s disease
16. Planned surgeries in the next 3 months
17. Enrolled in another HIV and/or substance use medication intervention study
18. Taking naltrexone in the past 30 days
19. Taking gabapentin in the past 30 days
20. Taking pregabalin in the past 30 days
21. Diagnosis of chronic obstructive pulmonary disease (COPD)

**2.7 recruitment goals**

We aim to randomize 45 participants into the trial and a total of up to 60 may be enrolled, but not randomized due to not meeting study entry criteria. Existing Russia ARCH (H-31200) participants will be offered to be screened for PETER PAIN. We will also screen St PETER (H-35288) participants at their final St PETER study visit for eligibility. Participants who have completed the LINC-II study (H-36706) will also be a potential recruitment source. Recruitment beyond PSMU will occur at local NGOs and Russia ARCH network hospitals including the major clinical HIV hospitals (Botkin Infectious Disease Hospital, Leningrad Regional AIDS Center, St. Petersburg City AIDS Center) and addiction hospitals (Leningrad Regional Center for Addictions, St. Petersburg City Addiction Hospital).

**2.7.a. sample size calculation and power**

The purpose of the pilot trial is to generate preliminary data for design of a future larger RCT to demonstrate efficacy. Thus estimation rather than testing is the main objective of this aim. However we provide illustrative power calculations for our primary outcome (change in pain severity) based on prior literature and the number of participants (15 in each arm) we intend to study. It is expected that 45 participants will be enrolled into the study and less than 15% will drop out by 8 weeks. We therefore assume 39 participants (13 per arm) will complete the 8-week follow-up. The following calculations assume 2-sided tests, with an overall significance level of 0.05. For the purposes of power calculations, we consider a simple, conservative setting based on a Bonferroni adjustment for multiple comparisons, although in our analyses we will use the Hochberg sequential correction method, an approach that will result in higher power than the Bonferroni method. To maintain an overall type I error rate of 5%, we assume each of the 2 primary pairwise comparisons (i.e. each intervention vs. placebo) will be conducted at an alpha level of 0.025 for the following power calculations. Based on the 2013 study by Younger, the standard deviation for change in pain severity was 2.9. We expect the standard deviation will be similar in the proposed study and the same for each randomized group. Given this assumption, the minimum detectable difference in change in pain severity between baseline and 8 weeks that the study can detect with 80% power is 3.8 for either of the 2 primary comparisons of interest (i.e., naltrexone vs. placebo; gabapentin vs. placebo), (e.g., -3.0 vs. 0.8 for naltrexone vs. placebo, respectively) using a 2-sided t-test. Because a main goal of the study is estimation of effect sizes for the design of a future study, we also estimate the width of the confidence interval for the mean difference between any two arms for the primary outcome. Our calculations assume 2-sided 95% confidence intervals. Assuming a standard deviation of 2.9 as above, the 95% confidence interval for the mean difference in change in pain severity for either intervention arm vs. placebo would be no wider than 4.7.

# **3. Intervention**

**3.1 intervention overview**

The study will randomize 45 HIV-positive persons with past year heavy alcohol consumption (by NIAAA definition of at risk drinking) and chronic pain (defined as bodily pain of moderate to severe intensity lasting for ≥ 3 months). Existing Russia ARCH participants, and past St PETER, LINC-II, and PETER PAIN participants will be offered to be screened for this study. Recruitment beyond First St. Petersburg Pavlov State Medical University (PSMU) will occur at local NGOs, Russia ARCH network hospitals including the major clinical HIV and addiction hospitals. Some participants may be screened over the phone, but all interested potential study participants will ultimately be invited for an in-person screening of eligibility at PSMU. After eligible participants are consented and enrolled, the RA will complete screening for eligibility by conducting a rapid HIV test for non-ARCH participants, a urine drug test, blood draw for AST/ALT and creatinine testing, and testing all women for pregnancy (urine). Once AST/ALT and creatinine results are received, participants will be notified by phone and if eligible, will be invited to return to Pavlov for their baseline visit.

At the baseline visit, participants will be administered the naloxone challenge and observed for signs of opioid withdrawal for 20 minutes. Naloxone blocks the effects of opioids. It works only if it is injected and lasts about 30 minutes. It is used in hospitals and by emergency services to reverse the effects of a heroin or other opioid overdose. It will cause withdrawal if injected into someone who is currently physically dependent on opioids and will be administered to make sure that the participant is not physically dependent on heroin or other opioids before s/he is given oral naltrexone. The naloxone challenge will only be administered if the information the participant provides to the staff indicates that s/he is not physically dependent on heroin or other opioids and if his/her urine drug test is negative. The challenge will be done by administering 0.8-mg naloxone slowly intramuscularly. The participant will be observed by a research assessor for signs of withdrawal for 5-20 minutes. If the participant experiences withdrawal after the naloxone test, his/her symptoms may be treated with 150mcg clonidine or 2mg of phenazepam orally and will go away in 45-60 minutes. As withdrawal symptoms precipitated by naloxone typically resolve within 20 minutes without treatment, use of these medications will be decided on a case-by-case basis by the treating Russian physician-investigator.

In the consent form, participants will be alerted that they may experience the following side effects if they need to be treated with clonidine or phenazepam:

The most frequent side effects (which appear to be dose-related) of clonidine are dry mouth, drowsiness, dizziness, constipation and sedation. The most frequent side effects of phenazepam are drowsiness, sedation, muscle weakness, and ataxia. These side effects generally decrease on continued administration and are a consequence of CNS depression.

The participant will not be started on the study medication until s/he passes a naloxone challenge, which means that s/he has no signs or symptoms of opioid withdrawal after naloxone is administered.

If the participant fails the naloxone challenge, s/he will be invited to come back within a week to repeat the challenge. Participants who pass the naloxone challenge will then complete the study assessment, cold-pressor testing and be randomly assigned to either low-dose naltrexone, gabapentin, or placebo. Study medication will be provided by trained physicians, who will instruct participants in proper medication administration and adherence.

Study participants, investigators, staff, and physicians administering the medications will be unaware of specific group assignment.

Participants will be treated for 8 weeks, and observed for 4 weeks post-treatment. Outcomes (pain severity and interference (BPI), biomarkers IL-6, 1β, IL-10, and TNF-α, cold pain tolerance, percentage of past month heavy drinking days, CD4 cell count, and HIV viral load) will be evaluated at the end of treatment.

Throughout the course of the study, participants will be expected to come in for eight in-person assessments. The schedule of study in-person visits/assessments are as follows: baseline, 1, 2, 4, 6, 7, 8, and 12 weeks. At week 4, we will assess for medication tolerability, discontinuation, treatment side effects, self-reported adherence to intervention medications, and pain (BPI) only. At week 8, we will assess pain (BPI), biomarker IL-6, IL-1β, IL-10, and TNF-α levels, cold pain tolerance using cold-pressor, pain interference, percentage of past month heavy drinking days, and HIV control (CD4 cell count & HIV viral load). At week 12 (post-treatment), there will be an assessment of pain (BPI) and cold-pressor test to assess whether pain reverts back to pre-treatment levels. Weeks 1, 2, 6, and 7 will be short visits, at which a brief assessment of tolerability, side effects, and adherence will occur, and we will ensure that participants have their medications (e.g. have not been lost or stolen).

Visits will be preceded by phone/text reminders to the participant or alternate contacts.

The text message will read: "This is a reminder that your visit to Pavlov Medical University is scheduled for_____at______. Please reply to confirm or call 973-53-96 to reschedule."

**3.2 randomization**

After consent and enrollment, participants will be randomly assigned to either low-dose naltrexone, gabapentin, or placebo with a 1:1:1 allocation ratio using permuted blocks to ensure equal numbers in each arm. Participants and research staff will be blinded to randomization arm.

Study medications will be obtained in St. Petersburg and delivered to the study pharmacist at PSMU prior to initiation of recruitment processes. The study pharmacist will deliver a supply of packaged boxes of study medication labeled with the group assignment to the study team.

Following completion of the baseline assessment, the RA will be directed to the electronic randomization screen in REDCap, which, once submitted, automatically assigns the participant to a randomization group. The RA then retrieves the pack of study medication from the identified group, labels the pack with the participant study ID, and enters the group number into REDCap, thus linking the two numbers.

3.3 intervention

Eligible participants will be randomly assigned into one of three study arms: 1) 4.5 mg naltrexone, 2) up to 1800mg gabapentin, or 3) placebo. Study medication will be provided by trained physicians, who will instruct participants in proper medication administration and adherence.

Drug capsules containing naltrexone hydrochloride (4.5mg), gabapentin (300 mg), and placebo will be prepared by NonStopPharma, a compounding pharmacy in St. Petersburg, Russia. Doses will look identical; participants and providers will be blinded to assigned medication. Participants will be instructed to take anywhere from one capsule per day to two capsules orally three times per day for 8 weeks. If a participant in any group experiences an adverse event, the dosage will be decreased by one capsule. Participants will be given 14 days’ worth of medications at baseline, 2-weeks, 4-weeks, and 6-week visits, with instructions to bring pill packs/leftover medications to the following visit.

The Food and Drug Administration (FDA) has indicated that an Investigational New Drug (IND) is not needed for studies conducted outside the United States.

3.3.a. Naltrexone

Participants randomized to this group will receive low-dose naltrexone for 8 weeks.

Participants will be instructed to take the medication from one to three times daily for 8 weeks. Each daily pill pack for participants in the low-dose naltrexone arm will contain one capsule of low-dose naltrexone (4.5mg) and the appropriate number of placebo capsules. Depending on the week, the number of placebo capsules per day will range between one and six, to match the dosage of the gabapentin arm. Naltrexone dosing will start on the day of the baseline visit, after successful passing of the naloxone challenge. If the participant does not pass the naloxone challenge, he/she will be brought back within a week to try again.

Medication will be packaged in pill boxes. Participants will receive 1 box of 7 capsules and 1 box of 21 capsules at baseline (14 days worth of medication), at the 2-week visit participants will receive 2 boxes of 42 capsules, at the 4-week visit participants will receive 2 boxes of 42 capsules; at the 6-week visit participants will receive 1 box of 42 capsules and 1 box of 33 capsules. In week 1, participants will take 4.5mg of naltrexone once daily. In week 2, participants will take 4.5mg of naltrexone once daily, and a placebo capsule twice daily. In weeks 3 through 7, participants will take 1 placebo capsule with 4.5 mg mg of naltrexone once daily, and 2 placebo capsules twice daily. In week 8, in days 1-4 participants will take 4.5 mg of naltrexone with a placebo capsule once daily and 2 placebo capsules twice daily; in days 5-7, participants will take 4.5 mg of naltrexone once daily, and a placebo capsule twice daily.

The most common side effects (reported in more than 1 in 10 people) of naltrexone are difficulty sleeping, anxiety, nervousness, abdominal pain/cramps, nausea and/or vomiting, low energy, joint and muscle pain, and headache.

Uncommon side effects (reported in less than 1 and 10 people, but more than 1 in 100 people) of naltrexone are loss of appetite, diarrhea, constipation, increased thirst, increased energy, feeling down, irritability, dizziness, skin rash, delayed ejaculation, decreased potency, and chills.

Rare side effects (reported in less than 1 in 100 people) of naltrexone include nasal congestion, itching, rhinorrhea, sneezing, sore throat, excess mucus or phlegm, sinus trouble, heavy breathing, hoarseness, cough, shortness of breath, nose bleeds, phlebitis, edema, increased blood pressure, non-specific ECG changes, palpitations, tachycardia, excessive gas, hemorrhoids, diarrhea, ulcer, painful shoulders, legs or knees; tremors, twitching, increased frequency of, or discomfort during, urination; increased or decreased sexual interest, oily skin, pruritus, acne, athlete's foot, cold sores, alopecia, depression, paranoia, fatigue, restlessness, confusion, disorientation, hallucinations, nightmares, bad dreams, eyes–blurred, burning, light sensitive, swollen, aching, strained; ears–"clogged," aching, tinnitus, Increased appetite, weight loss, weight gain, yawning, somnolence, fever, and dry mouth,

Please note that these side effects were reported among people taking 50mg of naltrexone. This study will use low dose naltrexone (4.5mg) and side effects are expected to be milder. In order to help prevent these adverse effects, participants will be instructed not to take more medication than what is provided by the study. Study clinicians will also be monitoring for signs of naltrexone overdose. These adverse effects are not expected to be likely.

3.3.b. gabapentin

Participants randomized to this group will receive gabapentin for 8 weeks.

Gabapentin dosing will start on the day of the baseline visit after successful passing of the naloxone challenge. If the participant does not pass the naloxone challenge, he/she will be brought back within a week to try again.

Participants will be instructed to take the medication from one to three times daily for 8 weeks. Medication will be packaged in pill boxes. Participants will receive 1 box of 7 capsules and 1 box of 21 capsules at baseline (14 days worth of medication), at the 2-week visit participants will receive 2 boxes of 42 capsules, at the 4-week visit participants will receive 2 boxes of 42 capsules; at the 6-week visit participants will receive 1 box of 42 capsules and 1 box of 33 capsules

Participants randomized to the gabapentin arm begin on a dose of 300 mg daily (300 mg qd). In week 2, participants will take 300 mg of gabapentin three times daily. In week 3 the dose will be titrated up to 1800 mg daily (300 mg+300 mg tid) will remain on the dose until week 8, when they will be tapered back down to 900 mg daily (300 mg tid). In week 8, in days 1-4 participants will take 1800 mg daily (300 mg+300 mg tid); in days 5-7, participants will take 900 mg daily (300 mg of gabapentin three times daily). Gabapentin will only be titrated up as tolerated by the participant. Participants will be monitored regularly for side effects and medication tolerability (weeks 1, 2, 4, 6, and 7) If a participant experiences adverse effects while on medication, the participant’s dose will be reduced down to the previous dose (or to a lower dose that the participant can tolerate). Research Assessors in this study are all trained addiction psychiatrists, with experience in clinical trials and adverse event monitoring.

The most common side effects (frequency of side effects cannot be estimated from available data) of gabapentin are lack of coordination, feeling tired, viral infection, fever, feeling drowsy, jerky movements, nausea and vomiting, difficulty with coordination, difficulty with speaking, double vision, tremor, unusual eye movement, and swelling (usually of legs and feet).

Rare side effects of gabapentin (reported in about 1 in 500) include suicidal thoughts or actions. These may include thoughts about suicide or dying, attempts to commit suicide, new or worse depression, new or worse anxiety, feeling agitated or restless, panic attacks, trouble sleeping (insomnia), new or worse irritability, acting aggressive, being angry, or violent, acting on dangerous impulses, an extreme increase in activity and talking (mania), other unusual changes in behavior or mood.

Other side effects (frequency of side effects cannot be estimated from available data) of gabapentin are serious or life-threatening allergic reactions that may affect one’s skin or other parts of the body such as the liver or blood cells. Symptoms may include skin rash, hives, difficulty breathing, fever, swollen glands that do not go away, swelling of the face, lips, throat, or tongue, yellowing of the skin or of the whites of the eyes, unusual bruising or bleeding, severe fatigue or weakness, unexpected muscle pain, frequent infections.

3.3.c. placebo

Participants randomized to this group will receive a placebo medication for 8 weeks.

Medication dosing will start on the day of the baseline visit after successful passing of the naloxone challenge. If the participant does not pass the naloxone challenge, he/she will be brought back within a week to try again.

Participants will be instructed to take the medication one to three times daily for 8 weeks. Medication will be packaged in pill boxes. Participants will receive 1 box of 7 capsules and 1 box of 21 capsules at baseline (14 days worth of medication), at the 2-week visit participants will receive 2 boxes of 42 capsules, at the 4-week visit participants will receive 2 boxes of 42 capsules; at the 6-week visit participants will receive 1 box of 42 capsules and 1 box of 33 capsules.

In week 1, participants will take 1 placebo capsule once daily. In week 2, participants will take 1 placebo capsule three times per day. In weeks 3 through 7, 2 placebo capsules three times per day. In week 8, in days 1-4 participants will take 2 placebo capsules three times per day; in days 5-7, participants will take 1 placebo capsule three times per day. The placebo medications will be composed of lactose and will not contain active ingredients. The placebo and active study medications will be indistinguishable by appearance and taste.

3.3.d. Naloxone challenge

At the baseline visit, participants will be administered the naloxone challenge and observed for signs of opioid withdrawal for 20 minutes. Naloxone blocks the effects of opioids. It works only if it is injected and lasts about 30 minutes. It is used in hospitals and by emergency services to reverse the effects of a heroin or other opioid overdose. It will cause withdrawal if injected into someone who is currently physically dependent on opioids and will be administered to make sure that the participant is not physically dependent on heroin or other opioids before s/he is given oral naltrexone. The naloxone challenge will only be administered if the information the participant provides to the staff indicates that s/he is not physically dependent on heroin or other opioids and if his/her urine drug test is negative. The challenge will be done by administering 0.8-mg naloxone slowly intramuscularly. The participant will be observed by a research assessor for signs of withdrawal for 5-20 minutes.

Discomfort during the naloxone challenge will occur if the participant is physically dependent on opioids. Naloxone can induce opioid withdrawal, such as feeling sick, stomach cramps, muscle spasms, feelings of coldness, heart pounding, muscular tension, aches and pains, yawning, runny eyes, and insomnia. If participants have withdrawal after the naloxone test, their symptoms may be treated with 150mcg clonidine or 2mg of phenazepam orally and are not anticipated to last more than 45-60 minutes. As withdrawal symptoms precipitated by naloxone typically resolve within 20 minutes without treatment, use of these medications will be decided on a case-by-case basis by the treating Russian physician-investigator.

The most frequent side effects (which appear to be dose-related) of clonidine are dry mouth, occurring in about 40 of 100 patients; drowsiness, about 33 in 100; dizziness, about 16 in 100; constipation and sedation, each about 10 in 100.

The following less frequent adverse experiences have also been reported in patients receiving clonidine tablets, but in many cases patients were receiving concomitant medication and a causal relationship has not been established: fatigue, fever, headache, pallor, weakness, and withdrawal syndrome. Also reported were a weakly positive Coombs test and increased sensitivity to alcohol, bradycardia, congestive heart failure, electrocardiographic abnormalities (i.e., sinus node arrest, junctional bradycardia, high degree AV block and arrhythmias), orthostatic symptoms, palpitations, Raynaud’s phenomenon, syncope, and tachycardia. Cases of sinus bradycardia and atrioventricular block have been reported, both with and without the use of concomitant digitalis, agitation, anxiety, delirium, delusional perception, hallucinations (including visual and auditory), insomnia, mental depression, nervousness, other behavioral changes, paresthesia, restlessness, sleep disorder, and vivid dreams or nightmares, alopecia, angioneurotic edema, hives, pruritus, rash, and urticaria, abdominal pain, anorexia, constipation, hepatitis, malaise, mild transient abnormalities in liver function tests, nausea, parotitis, pseudo-obstruction (including colonic pseudo-obstruction), salivary gland pain, vomiting, decreased sexual activity, difficulty in micturition, erectile dysfunction, loss of libido, nocturia, and urinary retention, thrombocytopenia, gynecomastia, transient elevation of blood glucose or serum creatinine phosphokinase, weight gain, leg cramps and muscle or joint pain, dryness of the nasal mucosa, accommodation disorder, blurred vision, burning of the eyes, decreased lacrimation, and dryness of eyes.

The most frequent side effects of phenazepam use are drowsiness, sedation, muscle weakness, and ataxia. These side effects generally decrease on continued administration and are a consequence of CNS depression.

Less frequent effects include vertigo, headache, confusion, depression, slurred speech or dysarthria, changes in libido, tremor, visual disturbances, urinary retention or incontinence, gastrointestinal disturbances, changes in salivation, and amnesia. Some patients may experience a paradoxical excitation, which may lead to hostility, aggression, and disinhibition.

Jaundice, blood disorders and hypersensitivity reactions have been reported rarely. Raised liver enzyme values have occurred.

**3.4 medication considerations**

3.4.a symptom monitoring

The study staff (research clinicians [addiction physicians with extensive experience performing pharmacotherapy trials]) will provide participants with the correct medication packages and advice on how to take the capsules. The staff will be trained to assess for adverse medication effects and will follow established protocols for identifying and monitoring any ongoing adverse events, including referral to treatment as appropriate. Study participants will be actively monitored for adverse events. Symptoms will be assessed every two weeks (and monitored more frequently, if necessary) by trained clinical staff, while the participants are administered study medications.  Processes have be set up to address any adverse event brought to the attention of study staff during the trial.

3.4.b. adherence

As one of the study outcomes, medication adherence will be assessed at each study visit using the following measures.

**Capsule Counts**

Participants will be instructed to bring any unused medication to each study visit post-baseline. The RA will count and record the number of remaining capsules. We will extrapolate the amount of medication taken and determine the measure of adherence.

**Self-Report**

Medication adherence will also be measured through self-report using the modified Adult AIDS Clinical Trial Group (AACTG) ART adherence questions.

***Pill Diary***

Participants will be asked to keep a daily written record (“daily diary”) of pain severity, medication adherence, and alcohol use.

**Adherence Aids**

During each study visit medication instructions will be reviewed and strategies for adherence discussed with each participant. Adherence plans will be individually tailored to each participant, depending on his or her reason for non-adherence.

Participants will also be encouraged to set a reminder in their phone to take the study medication three times each day.

3.4.c. medication disbursement

Medication inserts will be provided to participants at baseline.

Medication distribution will be as follows:

**Naltrexone:**

- 7 capsules/box, 1 capsule/day (Week 1)
  - Week 1: 4.5 mg qd (4.5 mg total)
- 21 capsules/box, 3 capsules/day (Week 2)
  - Week 2: 4.5 mg naltrexone qd, 1 placebo capsule bid
- 42 capsules/box, 6 capsules/day (Week 3-7)
  - Weeks 3-7: 1 placebo to be taken with 4.5 mg naltrexone qd, 2 placebo capsules to be taken bid
- 33 capsules/box, (Week 8)
  - Days 1-4 (6 capsules/day): 1 placebo to be taken with 4.5 mg naltrexone qd, 2 placebo capsules to be taken bid
  - Days 5-7 (3 capsules/day): 4.5 mg naltrexone qd, 1 placebo capsule bid
- Participants will receive 1 box of 7 capsules and 1 box of 21 capsules at baseline (14 days’ worth).
- Participants will receive 2 boxes of 42 capsules at week 2 (14 days’ worth).
- Participants will receive 2 boxes of 42 capsules at week 4 (14 days’ worth).
- Participants will receive 1 box of 42 capsules and 1 box of 33 capsules at week 6 (14 days’ worth).

**Gabapentin:**

- 7 capsules/box, 1 capsule/day (Week 1)
  - Week 1: 300 mg qd (300mg total)
- 21 capsules/box, 3 capsules/day (Week 2)
  - Week 2: 300 mg tid (900 mg total)
- 42 capsules/box, 6 capsules/day (Week 3-7)
  - Weeks 3-7: 2 capsules of 300 mg tid (1800 mg total)
- 33 capsules/box (Week 8)
  - Days 1-4 (6 capsules/day): 2 capsules of 300 mg tid (1800 mg total)
  - Days 5-7 (3 capsules/day): 300 mg tid (900 mg total)
- Participants will receive 1 box of 7 capsules and 1 box of 21 capsules at baseline (14 days’ worth).
- Participants will receive 2 boxes of 42 capsules at week 2 (14 days’ worth).
- Participants will receive 2 boxes of 42 capsules at week 4 (14 days’ worth).
- Participants will receive 1 box of 42 capsules and 1 box of 33 capsules at week 6 (14 days’ worth).

**Placebo:**

- 7 capsules/box, 1 capsule/day (Week 1)
  - Week 1: 1 placebo capsule qd
- 21 capsules/box, 3 capsules/day (Week 2)
  - Week 2: 1 placebo capsule tid
- 42 capsules/box, 6 capsules/day (Week 3-7)
  - Weeks 3-7: 2 placebo capsules tid
- 33 capsules/box (Week 8)
  - Days 1-4 (6 capsules/day): 2 placebo capsules tid
  - Days 5-7 (3 capsules/day): 1 placebo capsule tid
- Participants will receive 1 box of 21 capsules and 1 box of 42 capsules at baseline (14 days’ worth).
- Participants will receive 2 boxes of 42 capsules at week 2 (14 days’ worth).
- Participants will receive 2 boxes of 42 capsules at week 4 (14 days’ worth).
- Participants will receive 1 box of 42 capsules and 1 box of 33 capsules at week 6 (14 days’ worth).

Medication boxes will also be labeled. The medication labels will contain the following information:

FOR CLINICAL TRIAL USE ONLY

NALTREXONE/GABAPENTIN/PLACEBO

STORAGE: Don’t store medication above 25°C.

KEEP OUT OF REACH OF CHILDREN AND PETS.

Return all unused medication to the study site.

DATE OF MANUFACTURE:

SHELF LIFE:

3.4.d. lost or stolen study medication

If participants report lost or stolen medication, they will be provided with extra study medication. If participants report losing medication more than once, the study team will be alerted and the case discussed to determine a plan of action.

3.4.e. discontinuation of study medication

If as a result of study testing at week 4, a participant is found to have AST/ALT >10x normal, medications will be discontinued and the participants will be appropriately monitored for resolution of the abnormalities. If a participant has AST/ALT >5x normal as a result of study testing at week 4, participant will be rechecked at their 6-week visit to ensure levels do not increase to the threshold of discontinuation. Any participant with symptoms of acute hepatic failure (jaundice, dark urine) will have AST/ALT checked.

Those who discontinue medication will be followed and analyzed by intention to treat.

Participants found to be pregnant during the study will have their study medication discontinued, but will still be followed-up for the duration of the study. Participants who report pregnancy outside of study visits will be instructed to immediately discontinue their study medication and requested to come in for a confirmatory urine pregnancy test.

**3.5 Schedule of Data collection**

|  |  | **Screen** | **Baseline Visit** | **1 week visit** | **2 week visit** | **4 week visit** | **6 week visit** | **7 week visit** | **8 week visit** | **12 week visit** |
| --- | --- | --- | --- | --- | --- | --- | --- | --- | --- | --- |
| Screening | Screening Questions | X |  |  |  |  |  |  |  |  |
|  | Verification of HIV, non-pregnancy, urine tox, and ALT/AST | X |  |  |  |  |  |  |  |  |
| Enrollment | Sign Informed Consent | X |  |  |  |  |  |  |  |  |
|  | Complete contact information/verify numbers | X |  |  |  |  |  |  |  |  |
|  | Randomization |  | X |  |  |  |  |  |  |  |
| Laboratory | Clinical Values |  | X |  |  | X |  |  | X | X |
|  | Pregnancy Test | X | X | X | X | X | X | X |  |  |
|  | Urine Drug Test | X | X |  |  |  |  |  |  |  |
|  | AST, ALT | X |  |  |  | X | X (if indicated) |  |  |  |
|  | Creatinine | X |  |  |  |  |  |  |  |  |
|  | Cold Pressor Test |  | X |  |  | X |  |  | X | X |
|  | Naloxone Challenge |  | X |  |  |  |  |  |  |  |
|  | Blood Draw (IL-6, IL-1β, IL-10, TNF-alpha, CD4, HVL) |  | X |  |  |  |  |  | X |  |
| Assessment | Full Study Assessment |  | X |  |  | X |  |  | X | X |
|  | Short Assessment |  |  | X | X |  | X | X |  |  |
| Intervention | Symptom Management/Adverse Events |  | X | X | X | X | X | X | X | X |
|  | Provide Medication Instructions |  | X |  | X | X | X |  |  |  |
|  | Give Study Medication |  | X |  | X | X | X |  |  |  |
|  | Discuss Adherence |  | X | X | X | X | X | X | X |  |
|  | Assess Adherence |  |  | X | X | X | X | X | X |  |
| Other | Provide Resource Card |  | X |  |  |  |  |  |  |  |
|  | Compensate for Participation | X | X | X | X | X | X | X | X | X |
|  | Report Adverse Events |  | X | X | X | X | X | X | X | X |
|  | Complete Tracking Forms | X | X | X | X | X | X | X | X | X |

3.5.a. visit windows

Baseline Visit

- Window open: 1 day post screening
- Target date: 3 days post screening
- Window close: 28 days post screening
- Window length: 27 days

1 Week Visit

- Window open: 5 days post baseline
- Target date: 7 days post baseline
- Window close: 10 days post baseline
- Window length: 5 days

2 Week Visit

- Window open: 11 days post baseline
- Target date: 14 days post baseline
- Window close: 20 days post baseline
- Window length: 9 days

4 Week Visit

- Window open: 21 days post baseline
- Target date: 28 days post baseline
- Window close: 37 days post baseline
- Window length: 16 days

6 Week Visit

- Window open: 38 days post baseline
- Target date: 42 days post baseline
- Window close: 45 days post baseline
- Window length: 7 days

7 Week Visit

- Window open: 46 days post baseline
- Target date: 49 days post baseline
- Window close: 52 days post baseline
- Window length: 6 days

8 Week Visit

- Window open: 53 days post baseline
- Target date: 56 days post baseline
- Window close: 73 days post baseline

Window length: 20 days

12 Week Visit

- Window open: 74 days post baseline
- Target date: 84 days post baseline
- Window close: 94 days post baseline
- Window length: 20 days

**3.6 data sources**

3.6.a questionnaires

Questionnaires will be administered at baseline, 1-, 2-, 4-, 6-, 7-, 8-, and 12-week study visits. At weeks 4 and 8, assessments of medication tolerability, discontinuation, treatment side effects, self-reported adherence, and self-reported pain (BPI) will occur. At week 12 (post-treatment), there will be an assessment of self-reported pain (BPI) only. At weeks 1, 2, 6, and 7 brief assessment of tolerability, side effects, and adherence will occur.

3.6.b. urine

A pregnancy test will be administered by trained clinical research staff at screening to determine eligibility and at each study visit (except for 8- and 12-weeks).

Pregnant women will be excluded from the study due to some reports suggesting possible adverse events with study medications. Participants found to be pregnant will discontinue their study medication but will still be followed-up for the duration of the study.

Urine will also be checked at screening and baseline, as part of the naloxone challenge, to ensure that participants have not recently used opiates (as opioid receptor antagonists would be contraindicated as they could precipitate withdrawal). The urine drug test will test for a range of opioids, specifically opiates (products of the poppy plant – morphine, heroin) and synthetic opioids (specifically methadone). If new opioids appear on the scene in Russia, such as fentanyl, then we will begin testing for this, but currently that is not thought to be an issue.

3.6.c. blood

Blood will be collected at screening and the 4- week study visit to assess AST/ALT and/or Creatinine levels. Blood may also be collected at the 6-week study visit for liver function testing, if indicated at the participant’s 4-week study visit (see Section 3.4E).

Additional blood will be collected at baseline and the 8-week study visit to assess pro-inflammatory biomarkers, IL-6 IL-1β, IL-10, and TNF-alpha, as well as measures of HIV control, CD4 cell count and HIV viral load. Levels of these pro-inflammatory markers will be measured on blood samples collected using commercially available enzyme-linked immunosorbent assay kits (R&D Systems). Blood specimens will be processed and analyzed locally at the ImmunoBioService (IBS) under the direction of Dr. Sergei Selkvo. Per current Russia ARCH protocols, the local laboratory will collaborate with study consultant Dr. Russell Tracy who directs the Laboratory for Clinical Biochemistry Research (LCBR) at the University of Vermont, which specializes in biomarker testing in large population studies and clinical trials.

| **Test** | **Timepoint** |
| --- | --- |
| Red Tube (9 mL ) | |
| IL-6 | Baseline, 8-weeks |
| IL-10 | Baseline, 8-weeks |
| IL-1β | Baseline, 8-weeks |
| TNF-α | Baseline, 8-Weeks |
| Purple EDTA (4 mL) | |
| HIV RNA | Baseline, 8-Weeks |
| Green Top (4 mL) | |
| CD4 | Baseline, 8-Weeks |
| Red Top (4 mL) | |
| AST/ALT | Screening, 4-weeks, 6-weeks (if indicated) |
| eGFR (creatinine) | Screening |

**Unsuccessful Draw Protocol**

At baseline and 8-weeks, a blood draw will be considered successful if the EDTA purple top tube is at least half full, the green top tube is at least ¾ full, and the red top tube is full.

Participants with unsuccessful baseline blood draws will receive 1/3 (350 RUB) compensation at the initial attempt, 1/3 (333 RUB) at their second attempt, and the final 1/3 (350 RUB) at their third attempt. If the second attempt is successful, they will receive the remaining 2/3 (650 RUB) of the compensation at that time.

If the blood draw is unsuccessful at the 8-week study visit, the participant will be given partial (1/2, 750 RUB) compensation for completion of the assessment. The participant has 30 days to complete the blood draw attempt. Blood draw attempts will occur weekly (up to 4 times, the fourth attempt will correspond with the participant’s 12-week study visit). The remaining 1/2 (750 RUB) of the compensation will be divided between the 4 weekly attempts (187 RUB for each attempt). If the second or third attempts are successful, participants will receive the remaining 1/2 or 3/4 (375 RUB-561 RUB) at that time.

| Baseline | Timing of blood draw | Blood draw to take place prior to assessment; If blood draw is not successful, no assessment is conducted. |
| --- | --- | --- |
|  | Compensation | If blood draw is not successful, participant receives partial (1/3) compensation. |
|  | Window | 30 days after completion of screener |
|  | Final attempt | 3 weekly attempts; at final attempt provide full compensation. |
| Follow up*  (8-week visit only) | Timing of blood draw | If blood draw is not successful, proceed to assessment. |
|  | Compensation | Given partial (1/2) compensation for completion of assessment. |
|  | Window | 30 days after completion of assessment. |
|  | Final attempt | 4 weekly attempts; at final attempt provide full compensation. |

3.6.D. Cold-Pressor Testing

The cold-pressor test (CPT) is an experimental model that assesses cold pain threshold and tolerance. It is a research paradigm that has been used by researchers for decades, and has been utilized to measure enhanced sensitivity to cold pain (hyperalgesia). The investigators will use an automatic cooling water bath that keeps a constant temperature of 0–2 degrees Celsius. Participants are instructed to place their dominant arm in the cold water bath so that ice water covers the hand and approximately 10 cm of the forearm, to verbally indicate when they first perceive pain and, to rate their pain on a scale of 0-100 at its very worse, and when they can no longer tolerate the ice bath, to remove their arm from the water. The time from submersion to those events will be measured using a stopwatch. Cold pain threshold is measured as number of seconds to the initial perception of pain, and cold pain tolerance is measured as seconds until withdrawal of hand. Trials will be discontinued at 3 minutes since after this time pain severity diminishes as numbness sets in. This data will be collected at baseline, week 4, week 8, and week 12. Participants will be requested to refrain from over the counter analgesic use (non-steroidal anti-inflammatory pain medications or acetaminophen) for 24 hours prior to the study visit.

3.6.E. Daily Pain Diary

Participants will be asked to keep a daily written record (“daily diary”) of pain severity, medication adherence, and alcohol use.

# **4. Study Procedures**

**4.1 recruitment**

Former Russia ARCH, St PETER, LINC-II and PETER PAIN participants, who have agreed to be contacted for future studies, will be screened for the study. Current St PETER participants will also be screened for the study at their final St PETER study visit. Recruitment beyond these study populations will occur at local NGOs and Russia ARCH network hospitals including the major clinical HIV hospitals (Botkin Infectious Disease Hospital, Leningrad Regional AIDS Center, St. Petersburg City AIDS Center) and addiction hospitals (Leningrad Regional Center for Addictions, St. Petersburg City Addiction Hospital) through notification of patients and providers at these sites. At each of the recruitment sites flyers with information about the trial will be distributed to clinicians and peers working with HIV-positive patients. Flyers will provide a phone number (at Pavlov) for interested individuals to call to find out more about the study and undergo initial screening over the phone. On occasion, Pavlov research assessors may travel to screen participants at the affiliated clinical locations.

**4.2 screening**

Screening may take place over the phone or in-person. Verbal consent for screening will be obtained from all potential participants. Potential participants will be asked by a research assessor, either on the phone or in-person, their age, HIV infection status, past year alcohol consumption, chronic pain (moderate to severe intensity), and pregnancy status, confirm residence within 100km of St. Petersburg, and possession of a phone, and two contacts to assist with follow-up, and ability and willingness to comply with all study protocols and procedures. If the participant is not fluent in Russian, has a cognitive impairment resulting in inability to provide informed consent, reports breastfeeding, has known uncontrolled psychiatric illness (such as active psychosis), has current suicidal ideations, has known active tuberculosis or current febrile illness, has a history of hypersensitivity to naltrexone, gabapentin, or naloxone, has known liver failure, has a history of Reynaud’s disease, has ALT/AST levels >5x ULN, CrCl<60mL/min, is currently using (past week) illicit or prescribed opiates, is unwilling to abstain from opiates in during the treatment period, is currently using psychotropic or neuroleptic medications or has a history of seizure disorder, has any planned surgeries in the next three months, if the participant is enrolled in another HIV and/or substance use intervention study, or has taken naltrexone or gabapentin in the past 30 days then s/he will be deemed ineligible and chronic obstructive pulmonary disease. We will also collect information on participants’ average pain in the past week, pain interference with their enjoyment of life in the past week, and pain interference with their general activity in the past week. These data will be used to describe the sample, including those individuals who screen ineligible, but will not be used for assessing eligibility. After informed consent is signed, a confirmatory pregnancy test will be done for all female participants and urine drug test for all. Rapid HIV testing will be conducted, via an Alere Determine^TM^ HIV-1/2 visual read, qualitative immunoassay for the detection of HIV-1 and HIV-2 antibodies, for all non-Russia ARCH/St PETER/PETER PAIN UH2/LINC-II participants. Participants have the option of bringing in a recent (must be dated within 4 weeks of the screening visit) liver function test result to the screening visit to assess for eligibility, all other participants will have their blood drawn and sent for AST/ALT testing. Data collected on participants who screen out will be kept in order to have an accurate record of the rate of enrollment among those screened for participation and to be able to identify reasons why potential participants are ineligible. The data will not contain identifying information.

If a participant is screened over the phone, the participant will be re-screened in-person.

If a participant is not eligible during the phone screening or in-person screening, research assessors can re-contact the potential participant in the future to be re-screened. The decision to re-screen will be based on the cause of their ineligibility (i.e. HIV-negative individuals will not be re-contacted) and the likelihood that their eligibility will change (i.e. those who do not present a normal creatinine level at the first re-screening will not be re-contacted as kidney function will be unlikely to change). Decisions for re-screening will be clinically decided.

Participants’ screening IDs will be linked to their subject ID in a separate REDCap project. Contact information will also be collected on this REDCap project. Study data will be collected on REDCap and organized by either screening ID or study ID.

**4.3 informed consent**

Research assessors will conduct the consent process as well as obtain written consent. After eligibility and interest in enrollment is determined, an RA will administer and document the informed consent of the participant in a private location. The study will be explained to eligible participants who will be offered participation in the study. Research assessors will answer any questions the participants may have including risks, benefits and alternatives (including non-participation) to participation, and will provide written materials describing the study. If participants are unsure whether they would like to participate, they will be allowed any amount of time they need to consider participation in the study. If the participant is not able to make a decision on the day of the initial visit, s/he will be invited to contact the study team once they have made their decision, at which point s/he will be re-screened, if more than 3 days have passed since his/her initial screening. The written informed consent (in Russian), including the risks, benefits and alternatives, will be signed by the participant and the research assessor. As part of the informed consent process we will make it explicit to participants that their involvement in the study does not constitute medical treatment and that they will not receive any HIV medical care as part of the study. We will provide a handout with information on addiction and HIV treatment services to participants at the baseline visit. A copy of the informed consent will be provided to the participant and a copy will be maintained by the research team. The time the administration of the informed consent began, and the time the informed consent form was dated and signed by the participant will be captured in the study website. Potential participants will be informed that refusal to participate will not affect their medical care at PSMU in any way and they will be informed of their right to drop out of the study at any time.

**4.4 visit flow**

**Pre-screen (optional):**

- Screening questions administered over the phone

**Screening visit:**

- Screening Questions
- Informed Consent and Enrollment
- Conduct HIV, urine drug test, draw blood for AST/ALT and Creatinine testing (if recent test result not provided), and pregnancy testing
- Compensate participant, collect locator/contact information and check contact phone numbers
- Schedule return visit, if eligible
- Provide resource document, appointment reminder card

**Baseline visit:**

- Review and update locator/contact information, verifying new numbers, as necessary
- Conduct urine drug testing and pregnancy testing
- Administer naloxone challenge
- Administer assessment questionnaire
- Assess baseline symptoms
- Randomize participant
- Conduct cold-pressor test
- Draw blood for biomarker, CD4, and HVL testing
- Introduce study medication and instructions and develop an adherence plan
- Provide study medication
- Observe participant for 30 minutes after the participant takes the first dose
- Compensate participant; provide appointment reminder card, participation card, daily diary, and schedule next visit

**1-, 2-, 6-, and 7-week medication visit**:

- Review and update locator/contact information, verifying new numbers, as necessary
- Collect a urine sample to check for pregnancy (females only)
- Draw blood for ALT/AST testing, if indicated (6-week only)
- Administer a brief assessment of tolerability and side effects
- Assess medication adherence
- Provide study medication (2-week, 4-week, 6-week visits)
- Compensate participant and schedule next visit

**4-, 8-, and 12-week full study visit**:

- Review and update locator/contact information, verifying new numbers, as necessary
- Collect a urine sample to check for pregnancy (4-week only, females only)
- Assess medication adherence
- Administer assessment questionnaire
- Conduct cold-pressor test
- Draw blood for ALT/AST (4-week, and 6-week if indicated only) and biomarker, CD4, and HVL (8-week only) testing
- Provide study medication (4-week only)
- Compensate participant and schedule next visit (4- and 8-week only)

**4.5 quality assurance**

**Informed consent quality assurance**

The RA will review Informed Consent Forms (ICFs) for completeness with the participant present. Items to check will include, but are not limited to: responses/initials collected for all questions, correct version of ICF used, signed and dated by both subject and RA. Both the RA and project manager will review ICFs weekly for completeness.

**Assessment quality assurance**

During the assessment, if the participant provides conflicting answers or answers that did not make logical sense (either within the same section or between sections), the RA will gently try to help the participant arrive at more logical answers. However, the RA will not force the participant to change his or her answers. Certain quality assurance checks are built into the assessment. The system will flag any inappropriate responses and prevent the RA from continuing until the issue is resolved. The RA will never guess to correct a mistake. The only instance when a change can be made to the completed assessment is in the event that the RA is 100% certain that an error was made in data entry.

**4.6 compensation**

Participants will receive the equivalent of US $8 in goods or cash for the screening visit, as this will require the collection of blood for laboratory testing. Participants will receive $15 in goods or cash for their participation in baseline. Participants will receive the equivalent of US $8 in goods or cash for the short check-in visits at weeks 1 and 7, the equivalent of US $12 in goods or cash for 2-, 4- and 6-week visits, and the equivalent of $23 in goods or cash for 8- and 12-week visits, which will require the collection of blood for laboratory testing (8-week only) and cold pressor testing.

**4.7 Retention**

**Baseline visit:** Retention begins at baseline by ensuring that the participant enjoys the experience of participating in the study, by explaining the informed consent and what would happen in the study, and by collecting excellent contact information, including both the address where the participant is registered and the address where the participant is currently staying. Participants will be asked to provide contact information for 4-5 alternative contacts who may know their whereabouts. Alternative contacts can include friends, family members, and social workers. Participants will be asked if any of their friends are participating in the study and to include them as alternative contacts, if possible. Contact numbers must be verified by calling the numbers with the participant present, using the following script:

I am calling from Pavlov University. Your friend/relative [NAME] is here with me and just enrolled in a study. He/she has listed you as an alternative contact. We will only call you if we are having trouble reaching [NAME] to see if you can help us connect with them. Today I am just calling to confirm that this number is active.

If the alternate contact cannot be reached at the baseline visit, the RA will try to reach the contact again at the next in-person study visit. If the RA is unable to reach the contact at the following study visit, the participant will be asked to provide a different alternative contact. Participants will also be asked for their email address and membership to any social networking platforms.

**All visits:** Participants will be offered tea, coffee, water, and snacks at each study visit to make their experience in the research study more enjoyable.

RA will offer to help participants add the next scheduled study visit to the calendar in their phone and set a reminder in their phone.

**Follow-up visits:** Contact information for participant and alternatives will be reviewed and updated at every visit.

**Other strategies:** Participants will be contacted by telephone with appointment reminders and email, if one is provided. The study team will also utilize social networking to connect with participants. If participants are unable to be reached via phone, in addition to attempting to reach them via text messaging and email, participants will be sent private messages on Vkontakte (Russian social network) utilizing an existing standard script to remind them of their upcoming study visit. No sensitive information will be revealed or ascertained using this method. Study participants will be asked to contact the study team if their phone number changes between study visits; participants will be compensated 300 rubles in goods or currency for this information. All no-shows will be followed up to reschedule appointments.

Transportation will be arranged (i.e., a social taxi or Uber) for participants who are unable to come to First St. Petersburg Pavlov State Medical University due to a lack of available transportation.

# **5. Assessments**

**5.1 baseline assessment**

Participants will be assessed as part of this study using validated interview instruments covering the following topics:

- Demographics (marital status, education, employment, individual Income/security, date of birth, spouse HIV status, living situation)
- Alcohol use by the Timeline Followback (TLFB) calendar method with a 30-day assessment^4,5^
  - Participants will also be provided with a calendar to record daily alcohol use. They will be asked to complete this calendar at home and bring it back to all subsequent study visits. The calendar will be used as an aid by the RA when completing the TLFB with the participant.
- Alcohol Use Disorder using the AUDIT (baseline only).^6^
- Alcohol craving measured by the Penn Alcohol Craving Score^7^
- Depressive symptoms through the Center for Epidemiologic Studies Depression Scale (CES-D)^8^
- Anxiety by the Generalized Anxiety Disorder 7-item Scale (GAD-7)^9^
- ART use and adherence, using modified Adult AIDS Clinical Trial Group (AACTG) ART adherence questions^10^
- Co-morbidities by an adapted Veterans Aging Cohort Study patient questionnaire^11^
- HIV symptoms through a validated HIV Symptom Index from the NIAID Adult AIDS Clinical Trials Group
- Prescription and non-prescription medication use
- Brief Pain Inventory using a modified Short Form of the Russian Brief Pain Inventory^12^
- Adjunct treatments of pain
- Drug use and HIV Risk Behaviors by an adapted version of the Risk Behavior Survey^13,14^
  - Participants will be asked about lifetime use and past 30 days use.
- General health, quality of life, and cognitive function by the Veterans RAND 12-Item Health Survey (VR-12) and the Medical Outcomes Study HIV Health Survey (MOS-HIV)^15,16^

**5.2 follow-up assessments**

Content of assessments administered at the 4-, 8-, and 12-week visits will be subsets of the baseline assessment. Please see table of study questionnaires at the end of this section. Sections not administered at baseline, but administered at follow-up visits include:

- Medication adherence using modified Adult AIDS Clinical Trial Group (AACTG) ART adherence questions^10^
- Medication satisfaction via the Treatment Satisfaction Questionnaire for Medication (TSQM)^17^
- Medication tolerability using modified Adult AIDS Clinical Trial Group (AACTG) ART adherence questions^10^

5.2.a. medication visits assessments

Medication adherence and symptoms will be assessed at weeks 1, 2, 6, and 7.

|  |  | Treatment | | | | | | Post-Treatment |
| --- | --- | --- | --- | --- | --- | --- | --- | --- |
|  |  |  | | | | | |  |
| Administered Assessment | Baseline | 1-week | 2-week | 4-week | 6-week | 7-week | 8-week | 12-week |
| Demographics | X |  |  |  |  |  |  |  |
| 30-day Alcohol Timeline Followback (TLFB) | X |  |  | X |  |  | X | X |
| Alcohol Use Disorder (AUDIT) | X |  |  |  |  |  |  |  |
| Alcohol Craving | X |  |  | X |  |  | X | X |
| Depressive Symptoms (CES-D) | X |  |  | X |  |  | X | X |
| Anxiety (GAD-7) | X |  |  | X |  |  | X | X |
| ART Use & Adherence  (Adherence at baseline & 12-week only) | X |  |  | X |  |  | X | X |
| Co-Morbidities | X |  |  |  |  |  |  |  |
| HIV Symptom Index | X |  |  |  |  |  |  |  |
| HIV Risk Behaviors | X |  |  |  |  |  |  |  |
| Medications | X |  |  | X |  |  | X | X |
| Brief Pain Inventory | X | X | X | X | X | X | X | X |
| Self Management of Pain | X |  |  |  |  |  |  |  |
| Drug Use (modified RBS) | X |  |  | X |  |  | X | X |
| VR-12 Health Survey & MOS HIV | X |  |  | X |  |  | X | X |
| Baseline Symptom Monitoring | X |  |  |  |  |  |  |  |
| Follow-Up Symptom Checklist |  | X | X | X | X | X | X |  |
| Medication Adherence |  | X | X | X | X | X | X |  |
| Medication Satisfaction (TSQM) |  |  | X | X | X |  | X |  |
| Medication Tolerability |  | X | X | X | X | X | X |  |
|  |  |  |  |  |  |  |  |  |

# **6. Participant Safety**

Participant safety will be monitored every two weeks and more frequently, if necessary.

**6.1. specification of safety parameters**

An **Adverse Event (AE)** is defined as any untoward or unfavorable medical occurrence in a human subject, including any abnormal sign (for example, abnormal physical exam or laboratory finding), symptom, or disease, temporally associated with the subject’s participation in the research, whether or not considered related to the subject’s participation in the research.

An AE can therefore be any new sign, reaction, symptom, event, disease or a worsening in frequency or severity of a preexisting condition that occurs during the course of the study.

*Stable chronic conditions that were present prior to study entry and do not worsen are not considered AEs.*

**SERIOUS Adverse Event (SAE)** – for an event to be defined as serious, it will be Grade 1-6 below. Grade 0 would be “not serious”.

Grade (1) results in death;

Grade (2) is life-threatening (places the subject at immediate risk of death from the event as it occurred);

Grade (3) results in inpatient hospitalization or prolongation of existing hospitalization;

Grade (4) results in a persistent or significant disability/incapacity;

Grade (5) results in a congenital anomaly/birth defect; or

Grade (6) based upon appropriate medical judgment, may jeopardize the subject's health and may require medical or surgical intervention to prevent one of the other outcomes listed in this definition (examples of such events include allergic bronchospasm requiring intensive treatment in the emergency room or at home, blood dyscrasias or convulsions that do not result in inpatient hospitalization, or the development of drug dependency or drug abuse).

**Unanticipated Problem (UP)** – for an event to be an Unanticipated Problem it must

- be unexpected AND
- be related or possibly related to participation in the research AND
- suggest that the research places subjects or others at a greater risk of harm (including physical, psychological, economic, or social harm) than was previously known or recognized. OR meet the definition of SERIOUS

**Suspected Adverse Drug Reaction** – Any adverse event for which there is a reasonable possibility that the drug caused the adverse event. Reasonable possibility means there is evidence to suspect a causal relationship. It is considered unexpected if it is not consistent with the risk information described in the general investigational plan. A suspected adverse drug reaction will be defined as a recorded adverse event that is unexpected and deemed to be possibly, probably, or definitely related to the study drug.

**6.2 the methods and time for assessing, recording, and analyzing safety parameters**

- Participant symptoms will be assessed at baseline to document any chronic conditions or symptoms that existed prior to introduction of study medication. These will be documented on the Baseline AE log. This list will be reviewed and compared to reported events throughout the study. *If the participant reports the same ongoing symptom (same severity) during subsequent visits, the symptom should not be recorded as an Adverse Event (AE). If the event is new (not previously reported) or worsened, as determined by RA, then the AE should be reported.*
- During each scheduled visit, the RA will ask the participant how he or she feels and review the list of symptoms of concern (starting with the symptoms recorded at the previous visit). Any event that meets the above criteria for an AE/SAE/UP must be recorded. In the case of unresolved AEs, clinical staff will update the AE log with any follow-up information that is gathered during their investigation.
- The site will receive the results of all blood work that is performed on study participants from the designated lab. If the lab results meet the criteria described in the protocol as an AE and are considered clinically significant by the site clinician then an AE will be recorded. **See “Discontinuation of Medications” for severe hepatoxicity thresholds.**
  - Participants will be alerted of abnormal lab results and will receive a recommendation to see their local provider. All abnormal lab results obtained at the baseline visit will be listed on the Baseline AE log.
- All AEs will be assessed to determine if they meet criteria for a Serious Adverse Event (SAE). Jeffrey Samet, MD, MA, MPH and Judith Tsui, MD, MPH will be designated to distinguish a serious adverse event (SAE) from a non-serious adverse event. Drs. Evgeny Krupitsky, MD, PhD, D.Med Sci and Elena Blokhina, MD. PhD will monitor SAE and AE on site. If the AE is serious, then the SAE form must be completed and appropriate reporting measures followed (see below). Investigators are encouraged to consult with the US team, if they are uncertain how to classify an event.
- The list of subject’s current medications will be reviewed and updated at every study visit, starting at baseline.

The study will take place in a medical setting where standard procedures are in place to assist patients who experience acute events. If consequences arise due to research procedures (e.g. distress, anxiety, suicidal thoughts,) the physician investigators will be available to assess participants and make appropriate interventions or referrals based on the clinical circumstances. Any participant who voices current suicidality, is experiencing a psychiatric emergency, or expresses the intent to harm others during the assessments will be reported to an on-site physician, immediately. That physician will determine the appropriate course of action, which will depend on location of the event and the clinical situation. Patients will be escorted to receive care by appropriate staff if deemed necessary.

- If an event is discovered outside of the scheduled study visits, it must still be recorded accordingly.
- Action Taken will be determined by the RAs for all AEs that are Mild and Moderate (unless specified below) and by Dr. Krupitsky or Dr. Blokhina for SAEs and AEs that are severe, life-threatening or fatal.

**6.3. procedures for eliciting reports of and for recording and reporting adverse event and intercurrent illnesses**

For any reported side effect: While with the participant, study personnel will listen, identify, and document the symptoms. The following symptoms will be assessed at baseline and during medication check-ins and study visits while participant is taking the study medication. All events will be documented on the Symptom Checklist and on AE forms.

| **Symptom** |
| --- |
| Agitation and/or Irritability |
| Anger |
| Depressed mood |
| Anxiety (includes nervousness and panic attacks) |
| Insomnia and/or other sleep problems |
| Abnormal dreams and/or nightmares |
| Headaches |
| Dizziness/lack of coordination |
| Nausea and/or vomiting |
| Joint/muscle pain |
| Abdominal pain/cramps |
| Fatigue |
| Drowsiness |
| Difficult speaking |
| Tremor |
| Jerky movements |
| Fever |
| Double vision |
| Swelling (legs or feet) |
| Unusual eye movement |

6.3.a. other Events

- Other events may or may not be associated with study drug use, but will be recorded, AE form completed and Dr. Blokhina or Dr. Krupitsky will be notified immediately to address the report.
- Dr. Blohkina or Dr. Krupitsky will evaluate the reported symptoms using clinical judgment to determine if they are related to the study and if study medication should be adjusted or ceased.
- If Dr. Blokhina or Dr. Krupitsky determine that study medications should be ceased, study personnel will attempt to contact the participant as soon as possible.
- If applicable, staff can advise participants to contact their physician immediately or call emergency services.
- Participants will receive a card to provide to medical staff in the case of a hospitalization or emergency stating that they are involved in a research study and are randomized to one of two study medications or placebo.

6.3.B. adverse event reporting

The following information should be present to complete AE and SAE forms during the initial report (on the day of finding out about the event):

- Description of the event
- Date of onset and resolution (if known)
- Severity – based on established criteria: <https://ctep.cancer.gov/protocoldevelopment/electronic_applications/docs/CTCAE_v5_Quick_Reference_5x7.pdf> * See Box 1

**Box 1. Guidelines for Severity Grades**

*Research assessor will refer to the guide for unique clinical descriptions of severity for each AE, which will follow the general guideline below:

- Grade 1 Mild; asymptomatic or mild symptoms; clinical or diagnostic observations only intervention not indicated.
- Grade 2 Moderate; minimal, local or noninvasive intervention indicated; limiting age-appropriate instrumental activities ADL*.
- Grade 3 Severe or medically significant but not immediately life-threatening; hospitalization or prolongation of hospitalization indicated; disabling; limiting self-care ADL**.
- Grade 4 Life-threatening consequences; urgent intervention indicated.
- Grade 5 Death related to AE.

Not all Grades are appropriate for all AEs. Therefore, some AEs are listed with fewer than five options for Grade selection. Grade 5 (Death) is not appropriate for some AEs and therefore is not an option.

**Activities of Daily Living (ADL)**

*Instrumental ADL refer to preparing meals, shopping for groceries or clothes, using the telephone, managing money, etc.
**Self care ADL refer to bathing, dressing and undressing, feeding self, using the toilet, taking medications, and not bedridden.

- Assessment of expectedness (is the event anticipated in terms of nature, severity, or frequency) given (a) the research procedures that are described in the IRB protocol and informed consent document; and (b) the characteristics of the subject population being studied
- Assessment of relatedness to study drug
- Any actions taken

Following the initial report, additional information may need to be gathered to complete the AE and SAE forms and to evaluate the event for relatedness. This process may include obtaining hospital discharge reports, physician records, autopsy records or any other type of records or information necessary to provide a complete and clear picture of the SAE and events preceding and following the event.

- All adverse events (including serious adverse events) will be followed until the event is resolved, stabilized, or until the end of individual’s participation in the study. Study investigators and clinicians will determine a follow-up plan (i.e., frequency and type of follow-up) on a case-by-case basis based on their clinical judgment.

6.3.c. sae reporting

If the SAE is not resolved or stabilized at this time or new information becomes available after the SAE form is completed, the SAE form should be updated as soon as possible. Any changes or updates to the SAE form will need to be re-reviewed and re-authorized by the study clinician.

In some cases, the study clinician may be unsure upon first learning of an SAE whether it is study related and/or expected, because study staff are awaiting more complete medical records. In such cases, the study clinician should make his/her best estimate of relatedness and expectedness, understanding that these determinations can be updated later. When updating determinations at a later date, the rationale for the change should be included in the SAE narrative.

SAEs and unanticipated events which are considered “at least possibly related” during the treatment and follow-up phases will be reported to the local IRB and to NIAAA within 48 hours of knowledge of the SAE and all other SAEs and unanticipated events will be reported within the time period mandated by the local IRB as indicated below.

The site must actively seek information about the SAE until the SAE is resolved, stabilized or until the participant is lost to follow-up and terminated from the study.

To summarize: upon determining an Adverse Event is Serious, the following procedures should be followed:

• The study staff, while meeting/talking with the participant or person providing details on the event, will gather as much information about the event from the participant as possible and complete the appropriate forms.

• The completed AE and SAE forms will be reviewed by key personnel on the Pavlov team (e.g. Evgeny or Elena). Any relevant clinical documents (labs, physician notes) available at that time will be provided to key personnel on the Pavlov team (e.g. Dr. Krupitsky or Dr. Blokhina) within 24 hours of finding out about the event.

- - After initial notification, the SAE must be updated with any additional information.

All unanticipated problems must be reported to the US team immediately.

AEs and SAEs will be reported to the URBAN ARCH Data Safety Monitoring Board every six months and as needed.

**BUMC Reporting Guidelines:**

**Reporting to the Boston Medical Center and Boston University Medical Campus IRB**

| **Information** | **Description** | **Form to use** | **When to Report** |
| --- | --- | --- | --- |
| 1. Unanticipated Problem associated with a fatal or life-threatening event | A fatal or life-threatening event that qualifies as an Unanticipated Problem (event was unexpected *AND* related/possibly related *AND* suggests greater risk, see definitions above) | **Reportable Events and New Information**  *AND* | Within **2 days** of the PI learning of a **fatal** or **life-threatening** event |
|  |  | **Change Request & Amendments** addressing needed changes | As soon as practical |
| 1. Unanticipated Problem NOT associated with a fatal or life-threatening event | An event that qualifies as an Unanticipated Problem (event was unexpected AND related/possibly related AND suggests greater risk, see definitions above) – whether or not it is also an Adverse Event or Serious Adverse Event | **Reportable Events and New Information**  *AND* | Within **7 days** of the PI learning of event |
|  |  | **Change Request & Amendments** addressing needed changes | As soon as practical |
| 1. Safety Monitors’ Reports **with** recom­mended changes | DSMB reports, Data Monitoring Committee reports, Adverse Event Monitoring Committee reports, audit reports, etc. **with** recommendations for changes to the study | **Reportable Events and New Information**  *AND* | Within **7 days** of the PI receiving the recommendations |
|  |  | **Change Request & Amendments** addressing needed changes | As soon as practical |
| 1. Major Deviations | A violation of IRB requirements or an unapproved change in the research study design or procedures that may affect the participant's rights, safety or well-being, that may affect the reliability of the study data, or was the result of willful misconduct. | **Reportable Events and New Information** | Within **7 days** of the PI learning of the deviation |
| 1. Adverse Events that are NOT Unanticipated Problems | All Adverse Events and Serious Adverse Events (see definitions above) that do *NOT* qualify as an Unanticipated Problem (event was expected *OR* unrelated *OR* suggests no new risk). | **Continuing Review Submission**:   - Section 4 if a Data Safety Monitoring Board (DSMB) report - Section 5 if no DSMB (AE/SAE summary report - must include PI’s conclusion that the pattern of events does not suggest a greater risk of harm) | At the time of Continuing Review |
| 1. Safety Monitors’ Reports **without** recom­mended changes | DSMB reports, Data Monitoring Committee reports, Adverse Event Monitoring Committee reports, audit reports etc. **without** recommended changes to the study | Attached to **Continuing Review** **Submission** Section 4 (The most recent report must be attached) | At the time of Continuing Review |

**Pavlov Reporting Guidelines:**

| What Event is Reported | When is Event Reported |
| --- | --- |
| Fatal or life-threatening unexpected, suspected serious adverse reactions | Within 7 calendar days of initial receipt of information |
|  |  |
| Non-fatal, non-life-threatening unexpected, suspected serious adverse reactions | Within 15 calendar days of initial receipt of information |
|  |  |
| AEs and UPs | On a quarterly basis |

**6.4. the type and duration of the follow-up of subjects after adverse events**

All non-mild adverse events (including serious adverse events) should be followed until the event is resolved, stabilized, or until the end of individual’s participation in the study. Dr. Blokhina or Dr. Krupitsky will determine a follow-up plan on a case-by-case basis based on their clinical judgment.

**6.5. unblinding Protocol**

Participants may be unblinded if there is an urgent medical need, as determined by the clinician evaluating the participant. If a participant is unblinded, study medication may be discontinued.

The following are examples of events that may result in emergency unblinding:

-An SAE occurs that is thought to be most likely or definitely related to the study drug.

-An AE or SAE occurs and the clinician treating the patient concludes that knowledge of the treatment arm is necessary to determine the therapy provided to the patient.

-The study drug is accidentally ingested by a child.

Participant experiences an event that may require unblinding.

Dr. Krupitsky/Dr. Blokhina assesses if the event requires emergency unblinding.

Request for emergency unblinding sent to the Pavlov pharmacist; Dr. Samet or Dr. Tsui are alerted. Only Dr. Blokhina/Dr. Krupitsky receive unblinding information; it is not shared with assessors.

Unblinding not done

YES

NO

**6.6. data safety and monitoring board**

The DSMB is responsible for ensuring subject safety (by reviewing blinded and unblinded safety data on a regular basis and assessing the safety of study procedures) and for monitoring the overall conduct of the study. No interim efficacy analyses will be conducted.

The DSMB is an independent group advisory to the PIs and the NIAAA, and is required to provide recommendations about starting, continuing, temporarily suspending the trial until certain conditions are met, and stopping the studies. In addition, the DSMB is asked to make recommendations, as appropriate, about:

- Benefit/risk ratio of procedures and participant burden
- Selection, recruitment, and retention of participants
- Protocol violations and adherence to protocol requirements
- Completeness, quality, and analysis of measurements
- Amendments to the study protocol and consent forms
- Participant safety
- Notification of and referral for abnormal findings

The Board will be composed of three full members (inclusive of the DSMB Chair) and 1 adjunct member.

Full Members of the DSMB are:

1. Theodore Colton, ScD (Professor of Epidemiology and Biostatistics, BUSPH), Chair of DSMB
2. Josiah Rich, MD (Professor of Medicine, Brown Medical School)
3. Jesse Stewart, PhD (Associate Professor of Psychology, Indiana University-Purdue University Indianapolis)

This DSMB will meet every six months. In cases where Institutional Review Boards or the NIH require more frequent monitoring, procedures will be put into place to conform to those requirements. An agenda will be provided detailing the studies to be discussed. It is estimated that the meeting will be scheduled for 1.5 hours. Each protocol and data review meeting will consist of two sessions: Open Session and Closed Session. Communication in the interim will be as needed. Unscheduled meetings can be requested by any party with the responsibility of overseeing the study. Requests can be made to the DSMB Chair, PIs, or NIAAA officials. The Chair, in collaboration with the Admin Core or NIAAA, will schedule any unplanned meetings.

The DSMB will provide recommendations about temporarily suspending or stopping the study. No interim efficacy or futility analyses are planned because of the risk level of the study and because patient outcomes are very unlikely to be worse in the intervention group in the study. The study team, including the project manager, statistician, co-investigators and research associates, led by the PIs, will evaluate the progress of the study, including periodic assessments of data quality, participant recruitment, accrual and retention, and factors external to the study when interpreting the data, such as scientific developments or the new availability of proven clinical services that could have an impact on the safety of the participants, the performance of the study or the ethics of the study.

# **7.** **Data Management**

**7.1 data collection**

All study data will be captured electronically on netbooks via a secure, web-based data capture system with the exception of: TLFB data and daily pain diaries, which will be collected on paper calendars.

**7.2 quality control process**

Quality control measures will include: detailed and unambiguous specifications for completion of data forms, including rules for coding skipped questions and missing data, training of study staff responsible for data collection and built-in validation rules, error checks, and question skips for electronic data capture. All data, regardless of capture method, will be reviewed for logic, skip patterns, response ranges, and internal inconsistencies. The RAs will be queried monthly regarding any noted inconsistencies.

**7.3 data security and confidentiality**

Screening forms and most other research paperwork will not include the participant’s name; instead, a unique ID will be assigned to each person screened, and another number assigned to those who enroll.

Tracking information will be kept similarly. Computer data will be password protected, and accessible only to research associates needing the information for follow-up purposes.

Any documents with identifiable participant data will only be accessible to the Russian Co-Investigators (Drs. Krupitsky and Zvartau), the project manager, and the RAs who recruit and follow participants, and perform QC.

Boston University School of Public Health’s Biostatistics and Epidemiology Data Analytics Center (BEDAC) will design, develop and maintain the electronic data collection forms, participant and data tracking, and underlying SQL database systems, and implement procedures for data quality control, including multiple checks for entered data. Electronic data collection forms will be designed to read easily, have clear instructions, preprogrammed skip patterns, real-time range checks and internal logic to minimize missing data and result in “cleaner” data at capture.

Electronic research data will be entered directly into REDCap, which is maintained on secure BU servers. All central systems are secured physically behind two card-access doors with access to the primary door restricted to key personnel. Data will be kept indefinitely. The mastercode will be kept separate from any study data.

Assessment data will be collected directly onto computers by research assessors. Use of electronic data collection will obviate the need for double data entry and limit missing data through required fields. All data will be converted to SAS datasets and reviewed for logic, skip patterns, response ranges, and internal inconsistencies. The Boston and Russian teams will work together to resolve data issues.

**7.4 web systems**

The study will use REDCap. REDCap is a secure web application for building and managing online surveys and databases and will be used for screening and assessments purposes. Study forms will be completed according to the schedule below.

| **FORM** |  | |  | |  | | **STUDY VISIT** | | | | | | | |
| --- | --- | --- | --- | --- | --- | --- | --- | --- | --- | --- | --- | --- | --- | --- |
|  | **Phone Screen** | **Screen & Baseline Visit** | | | | **1 week visit** | | **2 week visit** | **4 week visit** | **6 week visit** | **7 week visit** | **8 week visit** | **12 week visit** | **As Needed** |
|  |  | **Screen** | | **Baseline** | |  |  |  |  |  |  |  |  |  |
| Pre-Screener | X |  | |  | |  | |  |  |  |  |  |  |  |
| Screener |  | X | |  | |  | |  |  |  |  |  |  |  |
| Consent and enrollment form |  | X | |  | |  | |  |  |  |  |  |  |  |
| Full assessment |  |  | | X | |  | |  | X |  |  | X | X |  |
| Short assessment (adherence) |  |  | |  | | X | | X |  | X | X |  |  |  |
| Baseline Event Form |  |  | | X | |  | |  |  |  |  |  |  |  |
| Medication visit checklist |  |  | | X | | X | | X | X | X | X | X |  |  |
| Symptom Monitoring Form |  |  | |  | | X | | X | X | X | X | X |  |  |
| Naloxone Challenge Form |  |  | | X | |  | |  |  |  |  |  |  |  |
| Contact info |  | X | | X | | X | | X | X | X | X | X | X | X |
| Contact log |  |  | |  | |  | |  |  |  |  |  |  | X |
| Baseline tracking form |  |  | | X | |  | |  |  |  |  |  |  |  |
| Follow-up tracking form |  |  | |  | | X | | X | X | X | X | X | X |  |
| Lab forms |  | X | | X | |  | | X | X | X |  | X |  |  |
| Study conclusion form |  |  | |  | |  | |  |  |  |  |  |  | X |
| AE/SAE form (paper and electronic form) |  |  | |  | |  | |  |  |  |  |  |  | X |
| Medication collection (paper form) |  |  | | X | | X | | X | X | X | X | X | X |  |

# **8. Statistical Analysis**

This study will use an intent-to-treat analysis that includes all participants according to their randomized assignment. Descriptive statistics will be calculated for variables at baseline and each follow-up time to assess whether there appear to be any differences across treatment arms.

The main focus of this aim is the assessment the impact of each intervention (naltrexone, gabapentin, or placebo) on the primary study outcome (i.e., the continuous variable change in pain severity from baseline to week 8).

**8.1 primary analyses**

Descriptive statistics and graphic displays of all outcome measures will be generated.

The main analysis evaluating the impact of each intervention on the primary study outcome (i.e., the continuous variable change in pain severity and pain interference from baseline to week 8) will use multiple regression models to estimate mean differences between arms along with 95% confidence intervals. The models will include indicator variables to represent study arm. The 2 primary comparisons of interest are between each intervention vs. placebo, i.e., naltrexone vs. placebo; gabapentin vs. placebo, and will adjust for the multiple comparisons using the Hochberg sequential test procedure.^18^ We will compare naltrexone vs. gabapentin but these tests will be of secondary interest. If the data are normally distributed, multiple linear regression models will be used. However, if the distribution of change in pain severity is skewed, transformations of the data will be performed (e.g., log transformation). If an appropriate transformation is not identified, a median regression model will be used. Secondary outcomes (e.g., changes in biomarkers, cold pain tolerance) are continuous variables and will be analyzed using the same approach as above.

# **9. REPOSITORY**

**9.1 Purpose**

Data will be collected for the purposes of HIV/alcohol research and placed in the existing Uganda Russia Boston Alcohol Network for Alcohol Research Collaboration on HIV/AIDS (URBAN ARCH) Consortium Sample Repository (H-28658). The data and sample repository is a major asset of the Consortium to be utilized by all investigators and critical to future cross-cohort studies. This repository currently includes plasma, serum, whole blood, and DBS samples from the Consortium studies and is managed by the URBAN ARCH Admin and BDM Cores. Some of the stored samples have pre-designated uses per the study protocol, while others are stored for future studies, which will be used only with IRB approval.

All data and samples collected as part of this study will be placed in the repository for future HIV research.

**9.2 Stewardship**

Drs. Samet and Krupitsky will hold responsibility for the repository and will oversee the storage, transportation, and usage of samples in the repository. Some of of the day-to-day management of the repository will be delegated to Natalia Gnatienko, and to the BUSPH Biostatistics and Epidemiology Data Analytics Center (BEDAC). BEDAC will directly oversee all of the stored samples, data and the web-based specimen tracking and inventory management system.

**9.3 Release Of Samples/Data**

Investigators who propose using the repository data or samples will follow IRB procedures set forth by their own institution and will provide documentation that such requirements were met to the PI of the BUMC IRB repository protocol (Dr. Samet) prior to release of data or samples. If an institution does not have an IRB, the Admin Core will rely of their existing system of review to evaluate that the study or request is appropriate or valid enough to be sending samples or data to. The system in place requests that all external investigators, regardless of whether or not that have an IRB, complete and return the URBAN ARCH Consortium Project Proposal Attestation Form, URBAN ARCH Consortium Project Proposal Form and the BUMC Non-Engagement in Human Subjects Research Agreement (if applicable).

All subject identifiers will remain at BMC/BU Medical Campus. Only coded samples/data will be given to other investigators/entities - BMC/BU Medical Campus investigators will maintain the master-code/key and will never release it to outsiders.

# **10.** **Staff Training**

All study staff will be trained on the study protocol, including administration of study medication, symptom monitoring, cold-pressor testing, blood draws, and participant assessment prior to initiation of recruitment and enrollment. Training will take place in-person in St. Petersburg and via webinars.

# **11.** **Study Contacts**

This study will be jointly directed by sharing responsibilities over the year among two PIs, each with distinct strengths applicable to this study. The PIs, Drs. Samet and Tsui, will provide overall scientific leadership for this study and are ultimately responsible for coordinating investigations and activities at the different sites and ensuring integrity of the scientific work.

Dr. Samet will serve as the contact PI and will be responsible for the financial management of the study and communication between NIH and the rest of the leadership team. He will provide specific expertise on alcohol use and, given his extensive Russia, HIV, alcohol research experience, will manage the implementation of the study in Russia. Dr. Tsui will bring content expertise in the intersecting relationships of substance use and pain, as well as methodologic expertise in pain measurement. She will oversee all the components of the study and guide its direction by working with the research team. Drs. Samet and Tsui will share responsibility in monitoring the conduct of the entire study and interpreting and presenting final outcomes.

**Assignment of Responsibilities for PIs**

| **Samet** | **Tsui** | **Responsibilities** |
| --- | --- | --- |
| Primary |  | Contact PD, responsible for communication with NIH and rest of Project Team |
| Joint | Joint | Shared responsibility and authority for leading and directing the project intellectually and logistically |
| Secondary | Primary | Providing expertise on questions on pain |
| Primary | Secondary | Providing expertise on alcohol use and managing trial implementation in Russia |
| Primary | Secondary | Budget, subcontract, and spending monitoring and approval |
|  |  |  |

In Russia, the study will be led by site PI Dr. Evgeny Krupitsky. The study will be managed by Dr. Elena Blokhina and Dr. Vetrova in St. Petersburg and Sally Bendiks in Boston.

References

1. Goodman CW, Brett AS. A Clinical Overview of Off-label Use of Gabapentinoid Drugs. *JAMA Intern Med*. 2019;179(5):695. doi:10.1001/jamainternmed.2019.0086

2. Mason BJ, Quello S, Goodell V, Shadan F, Kyle M, Begovic A. Gabapentin Treatment for Alcohol Dependence. *JAMA Intern Med*. 2014;174(1):70. doi:10.1001/jamainternmed.2013.11950

3. Springer SA, Altice FL, Herme M, Di Paola A. Design and methods of a double blind randomized placebo-controlled trial of extended-release naltrexone for alcohol dependent and hazardous drinking prisoners with HIV who are transitioning to the community. *Contemp Clin Trials*. 2014;37(2):209-218. doi:10.1016/j.cct.2013.12.006

4. Robinson SM, Sobell LC, Sobell MB, Leo GI. Reliability of the Timeline Followback for cocaine, cannabis, and cigarette use. *Psychol Addict Behav*. 2014;28(1):154-162. doi:10.1037/a0030992

5. Sobell, L.C. Sobell MB. Alcohol Timeline Followback (TLFB) Users’ Manual. In: *Addiction Research Foundation*. Toronto, Canada; 1995.

6. Babor TF, Higgins-Biddle JC, Saunders JB, Monteiro MG. *AUDIT. The Alcohol Use Disorders Identification Test: Guidelines for Use in Primary Care*.; 2001. https://apps.who.int/iris/bitstream/handle/10665/67205/WHO_MSD_MSB_01.6a.pdf;jsessionid=2D0203817A8A3BF869B92CBE29BE7AF3?sequence=1. Accessed February 1, 2019.

7. Flannery BA, Volpicelli JR, Pettinati HM. Psychometric properties of the Penn Alcohol Craving Scale. *Alcohol Clin Exp Res*. 1999;23(8):1289-1295. http://www.ncbi.nlm.nih.gov/pubmed/10470970. Accessed August 23, 2019.

8. Radloff LS. The CES-D Scale. *Appl Psychol Meas*. 1977;1(3):385-401. doi:10.1177/014662167700100306

9. Spitzer RL, Kroenke K, Williams JBW, Löwe B. A Brief Measure for Assessing Generalized Anxiety Disorder. *Arch Intern Med*. 2006;166(10):1092. doi:10.1001/archinte.166.10.1092

10. Chesney MA, Ickovics JR, Chambers DB, et al. Self-reported adherence to antiretroviral medications among participants in HIV clinical trials: The AACTG Adherence Instruments. *AIDS Care*. 2000;12(3):255-266. doi:10.1080/09540120050042891

11. Justice AC, P. Modur S, Tate JP, et al. Predictive accuracy of the veterans aging cohort study index for mortality with HIV infection: A north american cross cohort analysis. *J Acquir Immune Defic Syndr*. 2013;62(2):149-163. doi:10.1097/QAI.0b013e31827df36c

12. Kalyadina SA, Ionova TI, Ivanova MO, et al. Russian Brief Pain Inventory: validation and application in cancer pain. *J Pain Symptom Manage*. 2008;35(1):95-102. doi:10.1016/j.jpainsymman.2007.02.042

13. Weatherby NL, Needle R, Cesari H, et al. Validity of self-reported drug use among injection drug users and crack cocaine users recruited through street outreach. *Eval Program Plann*. 1994;17(4):347-355. doi:10.1016/0149-7189(94)90035-3

14. Needle R, Fisher DG, Weatherby N, et al. Reliability of self-reported HIV risk behaviors of drug users. *Psychol Addict Behav*. 1995;9(4):242-250. doi:10.1037/0893-164X.9.4.242

15. Wu AW, Revicki DA, Jacobson D, Malitz FE. Evidence for reliability, validity and usefulness of the Medical Outcomes Study HIV Health Survey (MOS-HIV). *Qual Life Res*. 1997;6(6):481-493. http://www.ncbi.nlm.nih.gov/pubmed/9330549. Accessed February 4, 2019.

16. RAND Health Care. HIV Cost and Services Utilization Study (HCSUS) implemented by the Agency for Health Care Policy Research (AHCPR) and RAND. https://www.rand.org/health-care/projects/hcsus.html.

17. Atkinson, MJ, Sinha, A, Hass, SL, Colman, SS, Kumar, R, Brod, M, Rowland C. Treatment Satisfaction Questionnaire for Medication (TSQM). 2004. https://eprovide.mapi-trust.org/instruments/treatment-satisfaction-questionnaire-for-medication.

18. Hochberg Y. A sharper Bonferroni procedure for multiple tests of significance. *Biometrika*. 1988;75(4):800-802.
